# Expression Profiling of a Recombinant Inbred Line Panel Identifies the *dod‐19* Gene as a Regulator of Ethanol Response Behaviors in *C. elegans*

**DOI:** 10.1111/acer.70432

**Published:** 2026-10-23

**Authors:** Marijke H. van Wijk, Mark G. Sterken, Elizabeth C. Quamme, Joost A. G. Riksen, Basten L. Snoek, Stefan J. S. van de Ruitenbeek, Laura D. Mathies, Andrew G. Davies, Jill C. Bettinger, Jan E. Kammenga

**Affiliations:** ^1^ Laboratory of Nematology Wageningen University Wageningen the Netherlands; ^2^ Department of Pharmacology and Toxicology Virginia Commonwealth University Richmond Virginia USA; ^3^ Theoretical Biology and Bioinformatics Utrecht University Utrecht the Netherlands; ^4^ Virginia Commonwealth University Alcohol Research Center Richmond Virginia USA

**Keywords:** acute functional tolerance, *Caenorhabditis elegans*, differential gene expression, expression QTL, natural variation

## Abstract

**Background:**

Variation in gene expression is hypothesized to be a major source of phenotypic variation underlying the liability for alcohol misuse. *Caenorhabditis elegans* ( *C. elegans* ) has proven to be a valuable model in the identification of genes that modify behavioral responses to alcohol. Genetic variation in wild strains represents allelic differences that are tolerated by natural selection pressures and, therefore, are more likely to model the allelic differences that are present in humans compared with laboratory‐generated mutations.

**Methods:**

We examined the influence of natural alleles on gene expression in response to ethanol by mapping eQTL in *C. elegans* . We used a recombinant inbred panel derived from four wild strains to examine the regulation of ethanol exposure‐induced gene expression. We performed differential gene expression analyses and mapped eQTL. Detailed examination of *trans‐*band_ChrV‐0:3 Mb_ included quantification of the behavioral responses to ethanol for top candidate genes in the *trans*‐band.

**Results:**

Ethanol exposure increased *trans‐*eQTL and decreased *cis‐*eQTL. We observed the largest shift in distantly regulated transcripts on the first three Mb on chromosome V that contained 355 *trans‐*eQTL in a single *trans‐*band. The eQTL group in this *trans‐*band was involved in processes implicated in alcohol use disorder and its gene ontology terms included unsaturated fatty acids, responses to stress and environment, and immune and defense responses. We validated this genomic region using an introgression line and identified *nhr‐131* and *dod‐19* as potential polymorphic modifiers of *trans‐*band_ChrV‐0:3 Mb_. We tested null mutations in *nhr‐131* and *dod‐19* and found that *dod‐19* is required for the normal development of acute functional tolerance (AFT) to ethanol.

**Conclusions:**

Natural genetic variation in these wild strains primarily affected *trans*‐regulated gene expression. Examination of *trans‐*band_ChrV‐0:3 Mb_ identified *dod‐19* as a candidate expression modifier gene that regulates the development of AFT to ethanol and further implicates membrane biology in this process.

## Graphical abstract

The acute level of response to ethanol is a liability factor in the development of alcohol addiction with a strong genetic component. This study used variation in ethanol‐responsive gene expression in natural populations of *C. elegans* with variable behavioral responses to ethanol to implicate specific genes in influencing the level of response. The gene *dod‐19*, encoding a membrane‐bound protein, resides in a region associated with differential gene expression, and *dod‐19*'s function is required for the normal acute response to ethanol.

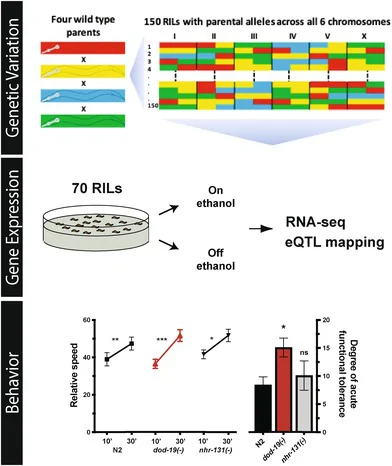

## 1 Introduction

Alcohol use is a major risk factor for the worldwide burden of disease (GBD 2016 Alcohol Drug Use Collaborators 2018). Individuals with Alcohol Use Disorder (AUD) have an impaired ability to stop or control alcohol use despite negative consequences. Roughly half of an individual's lifetime risk of developing AUD is determined by genetics (Schuckit and Smith 1996). One genetically influenced trait that correlates strongly with AUD risk is the physiological level of response (LR) to acute ethanol; individuals with a higher naïve LR to alcohol (ethanol, EtOH) are less likely to develop AUD (Heath et al. 1999; Salvatore et al. 2015; Schuckit et al. 2004). The genetic and molecular mechanisms underlying variation in LR and risk of developing AUD are incompletely understood.

Here we use the nematode *Caenorhabditis elegans* ( *C. elegans* ) to investigate variation in gene regulation upon ethanol exposure with the goal of identifying factors that affect LR. *C. elegans* is a suitable model organism to study the LR to EtOH because diverse physiological responses to EtOH can be observed and quantified, including initial sensitivity, and the development of acute functional tolerance, resulting in the detection of potential causal regulatory genes (Adkins et al. 2017; Davies et al. 2004, 2015; Mathies et al. 2015). We recently characterized gene expression in response to EtOH in the canonical lab wild‐type strain N2 and observed many similarities in differentially regulated EtOH response processes with other organisms, including humans (Sterken et al. 2021). Investigating gene expression in response to EtOH in *C. elegans* can enable us to learn more about potentially conserved biological processes that are involved in EtOH responses.

A widely established method for investigating gene expression regulation in response to EtOH is eQTL mapping (Kapoor et al. 2021; Zhou et al. 2022). In this approach, variation in gene expression levels is statistically linked to polymorphisms that segregate within a population. Transcripts that are regulated by local polymorphisms are presumed to be *cis‐*eQTL, whereas transcripts that are regulated by distant polymorphisms are *trans‐*eQTL. *Trans‐*eQTL can cluster together in *trans‐*bands that represent regions of the genome where one or multiple polymorphic modifier gene(s) regulate the expression of many distant genes; such modifiers can act as master regulator genes. Mapping eQTL has been widely applied in *C. elegans* and has yielded important information about shifts in gene expression regulation in multiple environments and genetic backgrounds (Snoek et al. 2021; Sterken et al. 2020).

To investigate the effects of naturally occurring alleles on variation in gene expression in response to EtOH exposure, we used a *C. elegans* multi‐parent recombinant inbred (mpRIL) panel. This panel was derived from four genotypically and phenotypically diverse founder strains (JU1511, JU1926, JU1931, and JU1941) and allows for more genetic variation and resolution than the classical two‐parent panels (Snoek et al. 2019; Volkers et al. 2013). We exposed 70 strains from the mpRIL panel to 0 mM and 400 mM EtOH for 4 h, characterized their transcriptomes, and mapped eQTL. This exogenous EtOH concentration yields tissue concentrations that are relevant to human alcohol use (Alaimo et al. 2012), and we chose this exposure paradigm based on previous time‐course experiments (Davies et al. 2015; Sterken et al. 2021). We found that EtOH exposure induces genotype‐specific gene expression changes that allowed us to map 176 *cis‐*eQTL, 2184 *trans‐*eQTL, and 5 *trans‐*bands. We confirmed the presence of the *trans‐*band on chromosome V‐0:3 Mb by generating an introgression line and analyzing its gene expression profile in response to EtOH. Using gene expression network analysis, we identified *nhr‐131* and *dod‐19* as candidate genes for affecting EtOH‐responsive gene expression. We reasoned that such genes may be important in EtOH response behaviors, and we found that a mutation in *dod‐19* in the N2 background caused a significant increase in the development of acute functional tolerance (AFT) to EtOH, indicating that the action of *dod‐19* is to inhibit AFT.

## 2 Materials and Methods

### 2.1 Nematode Strains and Growth Conditions

Animals were grown at 20°C on nematode growth medium (NGM) containing 2% agar to prevent burrowing into the agar plate and were fed with *Escherichia coli* OP50. The multi‐parent recombinant inbred line panel was previously generated by crossing their founders (JU1511, JU1926, JU1931, and JU1941) followed by repeated selfing (Snoek et al. 2019) (Table S1a). All populations were healthy and well fed for at least two generations before being tested.

The introgression line WN1200 was generated by backcrossing WN1158 x JU1941 for ten generations and selecting for the JU1931 introgression on the left arm of chromosome V; we ensured that the mitochondrial background of the final IL was from the JU1941 parent.

Before behavioral testing, both *dod‐19(ok2679)* and *nhr‐131(tm1376)* were backcrossed to N2 six times. We used PCR to follow the *ok2679* and *tm1376* genetic lesions through the crosses and to confirm the genotype before behavioral testing (Figure S9).

### 2.2 Selecting Populations for Transcriptomic Analysis

We used the published genetic map from Snoek et al. (2019) to calculate optimal selected sub‐populations capturing most of the genetic diversity within the mpRIL panel. The genetic diversity within a selected population was calculated as follows: pair‐wise correlations were made between all strains based on the available SNP map; the median correlation over the strain pairs present in the subset was taken as a measure of genetic diversity. We selected an optimal set by randomly drawing subsets of different sizes from the entire 200 mpRIL panel. Each subset contained 10 to 190 randomly drawn mpRILs, sampled in increments of 10 mpRILs. Each subset size was drawn 1 × 10^6^ times, resulting in 190 million populations in total. The genetic diversity within each subset was calculated. For each subset size, we took the lowest, closest to zero, and the highest correlating subsets, as well as the subset with the correlation that was closest to the median correlation of all 199 mpRILs. The selected subsets were used in a power analysis.

The statistical power for eQTL mapping in the mpRIL populations was determined in a power analysis (Sterken et al. 2017). In short, for each marker, random noise was introduced based on a standard normal distribution with sigma = 1, mu = 0. For each marker, eQTL were simulated that explained 20%, 25%, 30%, …, 80% of the variance on top of the randomly introduced noise. QTL were mapped using a single marker mapping based on the SNP distribution pattern (Snoek et al. 2019). We used a constant threshold of −log_10_(*p*) > 4.0 to determine the number of correctly detected QTL, the number of false positives, and the number of undetected QTL. This power analysis also includes the precision of the QTL location and the effect size estimation.

### 2.3 EtOH Exposure for Transcriptomic Analysis

We performed the EtOH exposures in batches. mpRILs were randomly assigned into six different batches. Each batch also contained each of the four founders as a control and reference (Table S1b).

We age‐synchronized the parent and mpRIL populations by allowing gravid adult hermaphrodites to lay eggs for 3 h, after which the adult hermaphrodites were removed. The resulting synchronized population was allowed to grow for 3 days at 20°C until they were first‐day adults.

Approximately 2000 age‐synchronized well‐fed young adult worms were washed off the culture plates with M9 buffer into 15 mL conical tubes, allowed to settle for 2 min, the supernatant was removed, the worms were washed with 5 mL M9, allowed to settle for 2 min, and the supernatant was removed. Each population was divided equally into two 15 mL conical treatment tubes with premixed M9 + 100% EtOH (0 mM or 400 mM EtOH). Worms were placed on a rotator at room temperature for 240 min. After the exposure, the worms were allowed to settle for 2 min, the supernatant was removed and the animals were washed twice with 5 mL M9, allowed to settle for 2 min, and the supernatant was removed. The worm pellets were transferred to a microfuge tube and spun for 1 min. The remaining supernatant was removed, and the pellets were flash frozen in liquid nitrogen. Samples were stored at −80°C until they were processed for RNA analysis.

### 2.4 RNA Isolation and Sequencing

RNA was isolated as described previously (Jovic et al. 2017; Sterken et al. 2021). In short, a Maxwell 16 AS2000 (Promega) instrument with a Maxwell 16 Simply RNA kit (Promega) was used. The manufacturer's instructions were followed with one modification: after the lysis step, 10 μL instead of 25 μL of a 20 mg/mL stock solution of proteinase K was added to each sample. Samples with the proteinase K were incubated for 10 min at 65°C in a ThermoMixer (Eppendorf) at 1000 rpm, after which the samples were cooled on ice for 1 min before adding them to the cartridge and resuming the standard protocol. RNA samples were stored at −80°C until further use. Out of 184 samples, 181 yielded sufficient RNA for library preparation.

RNA library preparation and sequencing was done by BGI on the DNBSEQ platform. Library preparation was done as follows: first, mRNA was enriched and purified by Oligo dT selection. The mRNA fragments were reverse transcribed, adaptors were ligated onto the fragments, and PCR was performed. PCR products were singled by heat separation and cyclized into a DNA nanoball which was sequenced. For each sample, at least 20 M clean paired‐end reads of 100 bp were generated. The raw reads can be found at ArrayExpress under E‐MTAB‐15535. Table S1b describes the design of the sequencing.

### 2.5 Alignment to Reference Genome, SNP Calling, Predicting Variant Impact, and Gene Filtering

Raw reads were aligned to the N2 reference genome (version PRJNA13758.WS282) using *hisat2 (version 2.2.1)* (Kim et al. 2019). Genome sorting and indexing was performed using *samtools (version 1.14)*. Genes that were located in hyper‐divergent regions were excluded from downstream analysis (Zhang et al. 2022). As a quality control we investigated gene expression correlation between the samples. Samples that had an average correlation of lower than 0.9 compared to the other samples were removed from further analysis. This excluded 4 of the 181 samples.

To select for genes with a detectable and reliable expression profile, we only selected genes that had an average transcript per million (TPM) value across all samples of > 1 in either the 0 mM or the 400 mM EtOH condition. All downstream analyses were done using the TPM expression values unless noted otherwise; downstream analysis for the ethanol response condition was done using the log_2_ ratio of the gene expression between 400 mM and 0 mM EtOH.

To confirm the expected genotypes, SNPs were called using *bcftools (version 1.14)* using the following parameters: *r* = V, d = 500, q = 30, Ou, *p* = 0.0011, an AD, and mv, and compared to the genetic map that we updated to the WS282 version (Snoek et al. 2019). One strain appeared to have been sequenced twice; therefore, we collapsed the two biological replicates. We manually checked and improved the genetic map that we used in all downstream analyses (Table S2).

The predicted effects of genetic variants on protein function were determined using *snpEff (version 5.0e)* (Cingolani et al. 2012; Mölder et al. 2021).

### 2.6 Principal Component Analysis

Gene expression values for each gene in each sample were normalized by dividing each sample's expression value by the mean expression of that gene across all samples, followed by log_2_ transformation (i.e., log_2_(expression/mean gene expression)). Genes with no detected expression in one or more samples were excluded, resulting in a dataset of 17,647 transcripts, representing 12,832 genes. The function *prcomp()* with *scale. = TRUE* in the statistical environment R (version 4.0.2) was used.

### 2.7 Differential Gene Expression Analysis

The R package *DESeq2 (version 1.30.1)* was used for analyzing gene expression differences between the samples (Love et al. 2014). For differential gene expression analysis, the raw gene expression counts of the genes that were filtered in the previous steps were used. To obtain a more accurate estimation of the effect sizes between the 0 mM and 400 mM ethanol exposure of all the mpRILs, the *lfcShrink()* function with type *apeglm* within DESeq2 was used (Zhu et al. 2019).

We compared differentially expressed genes identified in this study of natural variant‐derived strains with those reported by Sterken et al. (2021), which assessed the transcriptional response of the standard laboratory wild‐type strain N2 to ethanol exposure. The DEG dataset generated in this study was filtered using an adjusted *p* value threshold of FDR < 0.05 and separated into genes upregulated or downregulated in the EtOH condition. The Sterken et al. (2021) time‐course dataset was filtered to retain significant genes (FDR < 0.05) with available *C. elegans* gene identifiers and time points corresponding to late EtOH exposure (120 and 480 min). Overlapping upregulated and downregulated gene sets between the two studies were identified by comparing shared gene identifiers (Table S13). We also compared the DEGs identified in this study to those found in Mathies et al. (2026). In that study, N2 animals were exposed to EtOH for 18 h with and without a subsequent 6‐h withdrawal from EtOH (Table S14 (18‐h exposure) and Table S15 (18‐h exposure + 6‐h withdrawal)).

Differentially expressed genes (DEGs) identified in this study were compared with ethanol‐responsive DEGs reported in the meta‐analyses of Friske et al. (2025) and Huggett et al. (2026). (Table S16). For the *C. elegans* dataset, genes with an adjusted *p* value (FDR) < 0.05 were considered significantly differentially expressed.

For each mammalian species (human, non‐human primate, and rodent), significant ethanol‐responsive genes (FDR or adjusted *p* < 0.05, according to the original study) were extracted from the published supplementary datasets. Where multiple datasets were available for a species, significant genes were combined into a single species‐specific gene set. Orthologous relationships between mammalian genes and *C. elegans* genes were obtained using gProfiles. Genes lacking either a mammalian or *C. elegans* ortholog were excluded, whereas all identified orthologous relationships were retained for downstream analyses.

Species‐specific ortholog sets were compared with the significant *C. elegans* DEGs identified in the present study using shared *C. elegans* orthologs. The number of overlapping orthologous DEGs was determined for each species, and overlapping genes were further classified according to concordant transcriptional responses (i.e., genes upregulated in both species or downregulated in both species) based on the sign of the reported log_2_‐fold change.

Functional enrichment analysis was performed on the overlapping orthologous gene sets using gProfiler with default analysis settings. Gene Ontology terms were identified for the overlapping genes (Table S17), and Enriched Gene Ontology and pathway terms were ranked according to adjusted *p* value (Table S18).

### 2.8 Narrow‐Sense Heritability

The heritability of gene expression was determined with the package *heritability* using the function *marker_h2()* (version 1.3) which is based on a REML framework (Kruijer et al. 2015; Speed et al. 2012). The kinship matrix of the mpRILs was produced with the package *popkin* (version 1.3.13) and function *popkin()* based on biallelic SNPs (Snoek et al. 2019). For each gene, 100 permutations were performed to determine statistical significance. Here, TPM gene expression values were randomized over the strains and heritability was calculated.

### 2.9 eQTL Mapping, FDR, and Trans‐Band Calling

The eQTL were mapped using a single marker glm model:

$$E_{i,j}\;\sim G_{x,j}+e$$

where the gene expression *E* of gene *i* of mpRIL *j* is explained over the genotype *G* mpRIL *j* at marker location *x* plus an error term *e*. Mapping was done on the updated genetic map (Table S2). The FDR threshold was determined by permuting the entire data set for 10 times and was set at −log_10_(*p*) > 3.5; FDR = 0.05. A maximum of 1 eQTL per gene per chromosome was allowed. Confidence intervals were defined as the position where the drop in log_10_ odds was 1.5 compared to the peak. eQTL were classified as *cis* when the eQTL peak was within 1 Mb of the physical location of the gene. When eQTL mapped further away than 1 Mb, they were classified as *trans* (Snoek et al. 2021). The presence of *trans‐*bands was determined according to a Poisson distribution, where we tested if an interval of 1 Mb contained more *trans‐eQTL* than we could expect by chance (Rockman et al. 2010). The presence of a *trans‐*band was considered to be true when *p* < 0.0001.

The effect size of the eQTL was determined by:

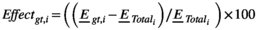

where *effect*_*gt,i*_ is the effect size of an eQTL per genotype, 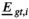 is the average gene expression of gene *i* per genotype *gt* (either JU1511, JU1926, JU1931, or JU1941), and 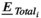 is the average gene expression of 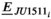, 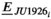, 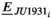, and 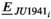. Pairwise comparisons of gene expression values for each genotype were performed with the function *glht()* with Tukey's method to adjust for multiple comparisons from the package *doBy* (version 4.6.7).

Clustering of *trans‐*eQTL per *trans‐bands* was done with K‐means clustering using the function *kmeans()* predetermining the number of clusters to be 4. All default parameters were used where max iterations was set on 10 and the algorithm used was Hartigan and Wong (1979).

### 2.10 Enrichment Analysis

GO‐term enrichment analyses were performed using g:Profiler using the ordered query function (Raudvere et al. 2019). The *EnrichmentMap (version 3.3.5)* tool in *Cytoscape (version 3.9.1)* was used for GO‐term enrichment comparison between datasets (Merico et al. 2010; Shannon et al. 2003).

### 2.11 Validation and Fine Mapping of Trans‐band_ChrV_ _‐0:3 Mb_

The parental strains JU1931 and JU1941, and the IL WN1200 were exposed to 0 mM and 400 mM EtOH (*n* = 6) and processed as described above. The genetic map was checked using the single nucleotide polymorphisms (SNPs) in the mapping data. Gene expression (TPM) of the genes that contained eQTL mapping to the *trans‐* band_ChrV‐0:3 Mb_ in the mpRIL population at 400 mM EtOH were filtered. The effect of the gene expression per gene per strain was calculated as described above. The correlation of the eQTL effect and the gene expression effect of IL WN1200 was calculated using the function *stat_cor()* from the *ggpubr* package.

To fine map the *trans‐*band polymorphic modifier(s), the *trans‐* band_ChrV‐0:3 Mb_ was divided into bins of 0.1 Mb and the correlation of the eQTL effect and the effect of IL WN1200 on gene expression was calculated as described above.

### 2.12 Prediction of Polymorphic Modifier Genes

Fine mapping the *trans‐*band_ChrV‐0:3 Mb_ resulted in a smaller genomic region; ChrV: 1.3–3 Mb. The top 20% (based on WN1200 effect size) of both the positively correlating genes and negatively correlating genes that were validated in the correlation analysis were filtered and used in further analysis. Multiple tools were used for predicting modifier genes that regulate the gene expression of the eQTL in *trans‐*band_ChrV‐0:3 Mb_. The validated, large effect size genes were submitted into the context‐centric prediction tool *WormNet V3* to find potential hub genes (Cho et al. 2014) and the *Wormexp* database to find mutants with a similar gene expression profile (Yang et al. 2016). The significance cut‐off for potential modifier genes in both tools was set at *p*.adj < 0.05. The validated, large effect size genes were compared with other eQTL datasets from N2xCB4856 RILs/RAILs to investigate whether there was a shared modifier polymorphism between the four parental strains and N2/CB4856 (Li et al. 2006; Rockman et al. 2010; Snoek et al. 2017, 2020; Sterken et al. 2017; Vinuela et al. 2010). Finally, mediation analysis was performed using the function *mediate()* from the *mediation* package (version 4.5.0) where we investigated if *cis‐* eQTL were mediating the gene expression of the validated, large effect size genes (Yimer et al. 2023). The genomic location and the polymorphic nature of all potential modifier genes were investigated. The potential modifier genes were then ranked.

### 2.13 Behavioral Assays

We assayed the behavior of animals carrying mutations in each of the two candidate genes, *nhr–131* and *dod–19*, as described in Davies et al. (2015). Briefly, we dried NGM plates without a bacterial lawn at 37°C for 2 h, weighed them to determine the volume of the media, melted 3 copper rings into the agar surface to act as corrals, and added 100% cold EtOH to a final concentration of 0 mM or 400 mM EtOH. The plates were then sealed with parafilm and the EtOH was allowed to equilibrate throughout the plate for 2 h at 20°C. Ten young adult hermaphrodites of each genotype (*nhr‐131(tm1376), dod‐19(ok2679)*, and N2) were moved to rings on unseeded NGM plates 30 min prior to the assay to allow them to acclimate to the food‐free conditions, and then they were transferred into corresponding rings on the assay plates and allowed to move freely. The movement of the animals on the assay plates was imaged with a CCD camera (Qimaging) in 2‐min movies beginning at 10 and 30 min of exposure. The speed of the animals was analyzed with *ImagePro‐Plus v6.0‐6.3* (Media Cybernetics). The average speed of all animals in a ring was used as the final value (10 animals per copper ring yielded (*n* = 1), we performed 6 biological replicates). N2 was used as a control to account for day‐to‐day and seasonal variation across assays. Each assay included 0 mM and 400 mM EtOH conditions performed in parallel; the data are presented as relative speeds (the speed on 400 mM EtOH/speed on 0 mM EtOH at each timepoint). All raw locomotion data are provided in File S1. Initial sensitivity is the effect of EtOH on locomotion speed at 10 min of exposure. The development of acute functional tolerance (AFT) is indicated by an increase in relative speed between 10 and 30 min. The extent of AFT was calculated by subtracting relative speed at 10 min from that at 30 min. Statistical comparisons were performed with two‐tailed paired Student's *t*‐tests (initial sensitivity and development of AFT, Figure 6 left panel) and unpaired two‐tailed *t*‐tests (comparison of levels of AFT, Figure 6 right panel) in Prism v. 9.5.1 (GraphPad).

## 3 Results

### 3.1 Natural Variation in Gene Expression in Response to EtOH

To characterize gene expression regulation in response to EtOH, we subjected our core population of 70 mpRILs, selected to effectively and efficiently sample the genetic diversity available in this population, along with their four founders (JU1511, JU1926, JU1931, and JU1941), to two conditions: 0 mM (control) and 400 mM EtOH (EtOH) exposure for 4 h, after which we isolated and sequenced mRNA. We chose 400 mM EtOH because this is a concentration that causes robust and reversible changes in several behaviors (Davies et al. 2003, 2004; van Wijk et al. 2023), and because we have previously shown that there is significant natural variation in the acute responses of the RILs to this concentration of EtOH (van Wijk et al. 2023). Following the mapping of raw reads and rigorous quality control, we focused our analyses on 17,647 transcripts representing 12,832 unique WormBase IDs.

To explore overall similarities and differences among samples, we conducted a Principal Component Analysis (PCA) (Table S3). PCA is an unsupervised statistical method that reduces complex, high dimensional data into a smaller number of components that capture the main patterns of variation in the dataset. The first two principal components collectively accounted for 33.7% of the dataset's variance (Figure 1A). Notably, the variance in these components reflected inherent gene expression disparities between genotypes, as illustrated by the distinct clustering of JU1941 in the PCA, setting it apart from the other three parents (Figure 1D). Principal Component 3, explaining 3.9% of the variance, highlighted the influence of EtOH on gene expression (Figure 1B). Additionally, Principal Component 5 indicated variance attributable to EtOH exposure, with treated animals exhibiting greater variability than control animals (Figure 1C). Our PCA analysis led us to conclude that genetic variation among the mpRILs, followed by EtOH treatment, predominantly contributes to the observed gene expression variation in this dataset.

**FIGURE 1:**
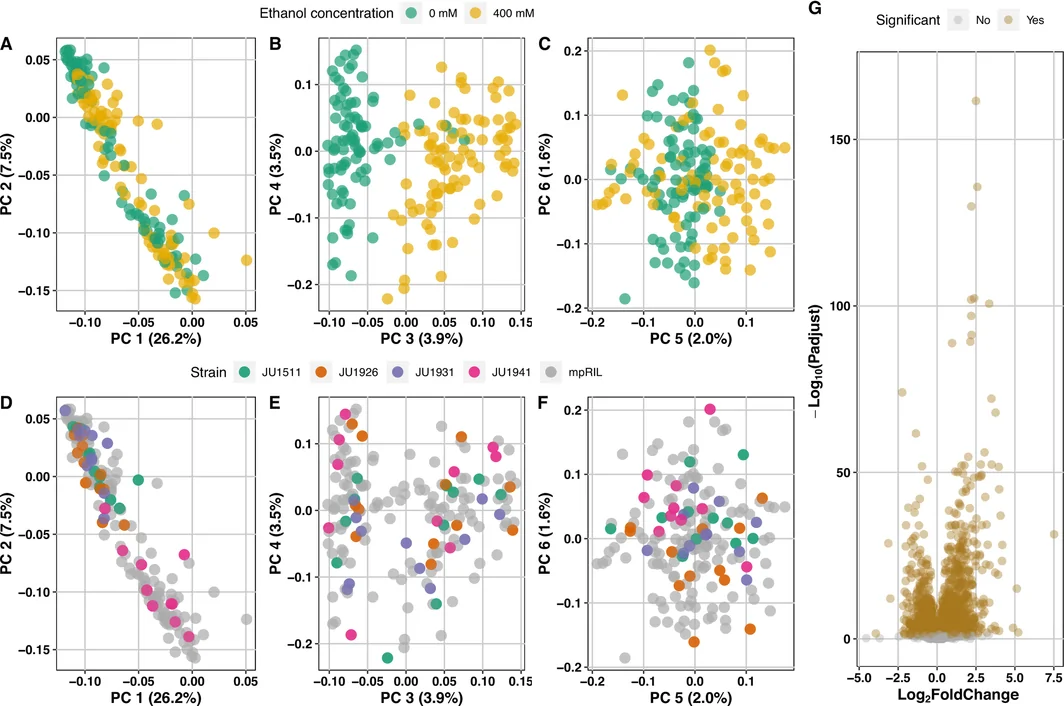
Gene expression variation is influenced by EtOH and genotype. (A–C) Principal component (PC) analysis of all the samples in the data. Each dot represents a single sample. The colors indicate the ethanol treatment (0 mM or 400 mM). PC 1 and 2 capture the variance that can be attributed to genotype. The EtOH effect is captured by PC 3 and PC 5. (D–F) Principal component analysis of all samples. Each dot represents a single sample. The colors indicate the strain, either parental (colors) or mpRIL (gray). PC 1 and PC 2 capture the variance that can be attributed to genotype. (G) Differential gene expression analysis between 0 mM and 400 mM EtOH exposure, including all mpRIL and parental samples. This analysis resulted in 1179 upregulated and 683 downregulated genes in response to EtOH (FDR < 0.05).

To examine the effect of EtOH on gene expression, we conducted a comprehensive differential gene expression analysis between 0 mM and 400 mM EtOH treatment across all samples. This analysis identified 1862 genes displaying differential expression between the two treatments, with 1179 upregulated and 683 downregulated in the EtOH condition compared to the no‐EtOH control condition (FDR < 0.05) (Figure 1G; Table S4). Gene Ontology (GO) term enrichment of the DEGs found in our current study revealed that the downregulated genes were notably associated with tRNA processes, whereas the upregulated genes exhibited strong associations with metabolic pathways and reproduction (Tables S5 and S6; Figure S2). These findings align with previous gene expression studies in response to EtOH in *C. elegans* , affirming the robustness of our experimental approach (Kwon et al. 2004; Sterken et al. 2021).

We asked how generalizable the EtOH‐responsive transcriptional response of natural variants is when compared to that of the standard laboratory wild‐type N2 strain. Sterken et al. (2021) examined the EtOH‐responsive transcriptional response of N2 treated with 400 mM EtOH, so we performed a direct comparison between findings in these two studies. Of our DEGs, we identified 414 shared upregulated genes and 152 shared downregulated genes between these studies. This indicates that there are both genotype‐specific and general components of the EtOH‐responsive transcriptional signature in *C. elegans* (Table S13).

We expect that the physiological response to EtOH will have commonalities across organisms. To evaluate the conservation of ethanol‐responsive transcriptional changes across phyla, we compared the *C. elegans* DEGs identified in this study with significant ethanol‐responsive genes reported in recent human, non‐human primate, and rodent meta‐analyses, using the *C. elegans* orthologs of the non‐worm genes in these studies (Friske et al. 2025; Huggett et al. 2026). Of the 1862 significant *C. elegans* DEGs that we identified, 309 overlapped with human ethanol‐responsive genes, 303 with non‐human primate DEGs, and 218 with rodent DEGs (Table S16). Among the overlapping genes, a substantial proportion showed concordant expression changes between species. Specifically, 129 human‐ *C. elegans* , 127 non‐human primate‐ *C. elegans* , and 116 rodent‐ *C. elegans* orthologs were upregulated in both compared species, whereas 57, 69, and 57 orthologs, respectively, were downregulated in both species (Table S16).

Functional enrichment analysis of the overlapping ethanol‐responsive orthologs revealed highly similar biological themes across the three cross‐species comparisons (Table S17). Among Gene Ontology Biological Process (GO) terms, *epithelium development* was enriched in all comparisons, whereas additional developmental and transport‐related processes were identified in human and rodent comparisons. Gene Ontology Cellular Component (GO) enrichment was remarkably consistent across species, with intracellular structures, organelles, and cytoplasmic components representing the most significantly enriched categories. Likewise, Gene Ontology Molecular Function (GO) analysis consistently identified small molecule binding, ion binding, and catalytic activity as the predominant molecular functions. At the pathway level, KEGG analysis identified *Metabolic pathways* as the only pathway enriched in all three comparisons, whereas *Fatty acid metabolism* (monkey) and *Cytoskeleton in muscle cells* (human) were uniquely enriched in individual species comparisons. Together, these results indicate highly similar functional processes associated with the transcriptional response to ethanol between species.

### 3.2 Gene Expression in Response to EtOH Is Heritable and Mainly Regulated *in Trans*

Next, we asked about the interplay between genetics and gene expression by computing narrow‐sense heritability (h^2^) for each expressed gene. In addition to assessing heritability under control and EtOH conditions, we extended our analysis to include heritability for EtOH responsiveness. This EtOH‐responsive dataset was generated by calculating the log_2_ gene expression ratio between the EtOH and control conditions, characterizing the upregulation or downregulation of genes in response to EtOH. Our findings revealed significant heritability in 2032, 2749, and 1152 genes for the control, EtOH, and EtOH response conditions, respectively (per gene FDR < 0.05) (Figure 2A; Table S7). Strikingly, 63% of genes exhibited heritable expression in only one of the three treatments (Figure 2B). This leads us to conclude that, for many genes in this population, their gene expression is subject to genetic regulation in a condition‐dependent manner.

**FIGURE 2:**
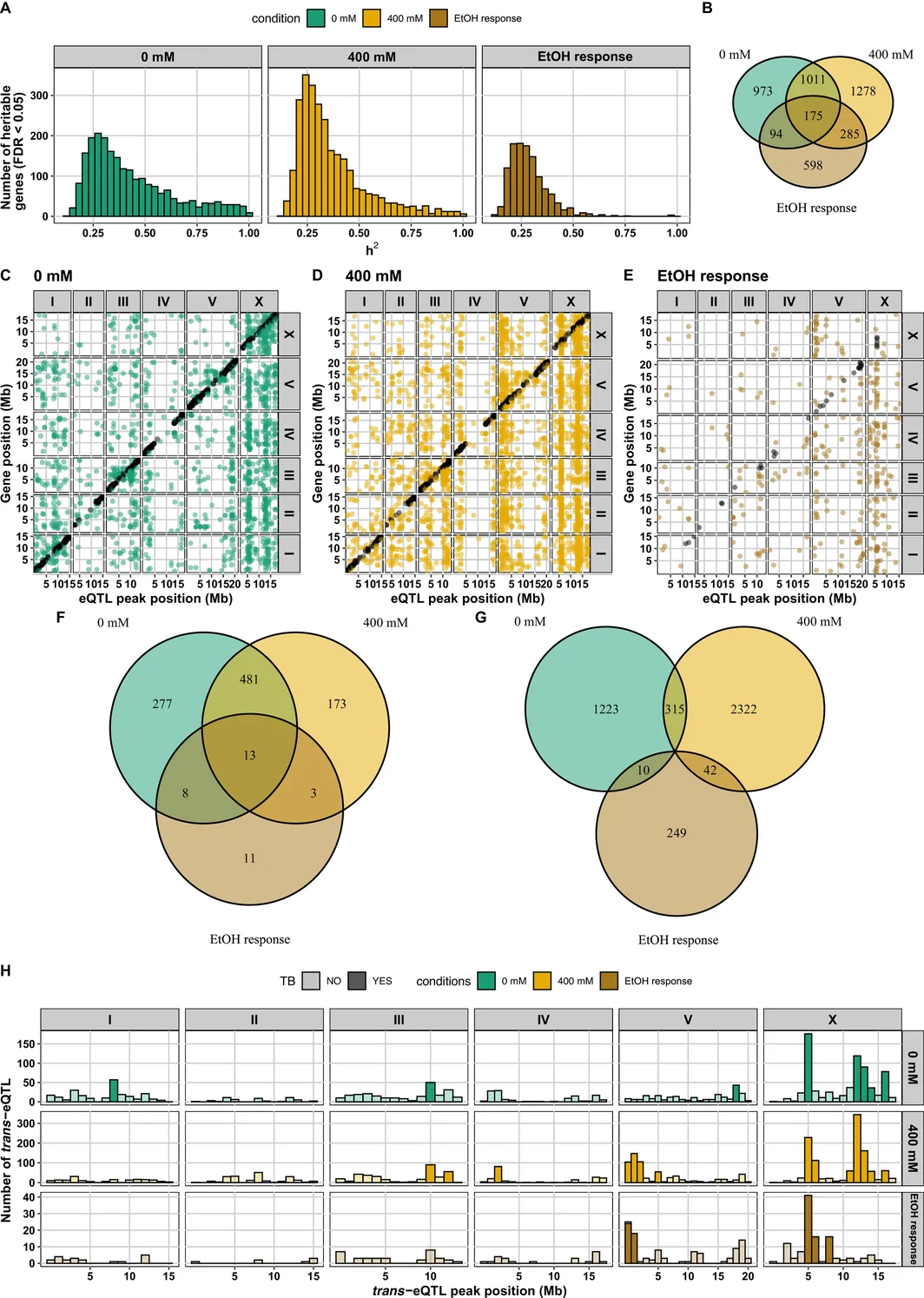
Differences in gene expression regulation between the 0 mM and 400 mM EtOH, and 0 mM versus 400 mM (EtOH effect) conditions. (A) We observed significant heritability for 2032 genes in the 0 mM EtOH condition, 2749 genes in the 400 mM condition and 1152 genes in the EtOH response condition (FDR < 0.05). (B) Genes that were significantly heritable overlapped, but most were only heritable in one condition. (C–E) Results of the eQTL mapping in the 0 mM EtOH condition, 400 mM EtOH condition, and EtOH response condition (−log_10_(*p*) > 3.5). Every dot represents an eQTL of a single transcript. Dots in black are *cis*‐eQTL that are mapped within 1 Mb of the physical location of the gene, dots in color are *trans*‐eQTL that are mapped outside a window of 1 Mb around the physical location of the gene. The *y*‐axis represents the physical location of the gene and the *x*‐axis represents the eQTL peak. (F) Overview of the *cis*‐eQTL mapped in the three conditions. High overlap of *cis*‐eQTL between the 0 mM and the 400 mM EtOH conditions, confirms that *cis*‐eQTL are generally robust between environments. (G) Overview of the *trans*‐eQTL mapped in the three conditions. *Trans*‐eQTL seem to be less common between conditions. *Trans*‐eQTL were considered to be overlapping when the eQTL in multiple conditions were *in trans* and on the same chromosome. (H) Similar and distinct trans‐bands between the conditions can be observed. The presence of a trans‐band was calculated by the Poisson distribution in which a bin of 1 Mb needed to contain more eQTL than could be expected by chance (*p* < 0.0001). Note that the y‐axes of the different treatments are different in scale.

To investigate the genetic regulation of gene expression, we conducted eQTL mapping in the control, EtOH, and EtOH response conditions (Figure 2C–E). In total, we identified 6025 eQTL (FDR < 0.05), with 2333 in the control condition, 3357 in the EtOH condition, and 335 in the EtOH response condition (Tables 1 and S8; Figure 2C–E). Although eQTL in both control and EtOH conditions were enriched for similar processes such as stress and immune responses, only eQTL in the EtOH condition exhibited enrichment for unfolded protein responses, a process intricately linked to EtOH responses in humans (Boukli et al. 2010). Notably, exposure to EtOH resulted in a ~73% increase in *trans‐*eQTL compared to the control condition (Table 1; Figure 2F,G). Additionally, *trans‐*eQTL displayed less overlap between the three conditions compared to *cis‐*eQTL, underscoring the profound influence of EtOH on distant gene regulation (Figure 2F,G). *Trans‐*eQTL were often clustered in specific genomic regions known as *trans‐*bands, reflecting the significant influence of a polymorphic modifier on the gene expression of numerous genes. A total of six *trans‐*bands were mapped in the control condition, eight in the EtOH condition, and three in the EtOH response condition (Figure 2H). Although most *trans‐*band locations overlapped between conditions, the number of eQTL differed. For instance, the *trans‐*band on chromosome X at 11:17 Mb contained 54 eQTL that were shared between the control and EtOH conditions, yet the total number of *trans‐*eQTL for the control and EtOH conditions were 323 and 337, respectively. This highlights that even when *trans‐*band regions overlap, the genes with eQTL mapping to those regions are largely distinct.

**TABLE 1:** Overview of the *cis*‐ & *trans*‐eQTL mapped in each condition.

Mapping condition	*cis‐*eQTL	*trans‐*eQTL	Total eQTL
0 mM EtOH	779	1554	2333
400 mM EtOH	670	2687	3357
EtOH response	35	300	335

To decipher gene expression patterns within each *trans‐*band, we employed k‐means clustering to group genes based on their genotype‐specific gene expression regulation (Figures 3 and S3–, S5; Table S9). For each *trans‐*band and eQTL, we calculated the effect of the physical genotype at each *trans‐*band bin of 1 Mb on transcript abundance (Figure 3A). Positive effects indicated relative upregulation, whereas negative effects indicated relative downregulation compared to the mean of the four underlying genotypes (Figure 3A). Our observations revealed that gene regulation could be primarily driven by one or two genotypes, but some *trans‐*bands exhibited more intricate regulatory patterns (Figures 3B–D and S3–, S5). For example, the *trans‐*band_ChrX‐13:14 Mb_ appeared to be regulated by the JU1931 genotype (Figure 3B). In *trans‐*band_ChrV‐2:3 Mb_, present in the EtOH condition, two gene clusters were observed, showing distinct phenotypes based on the presence of JU1926 or JU1931 genotypes (Figure 3C). Complex genetic regulation patterns were also evident in *trans‐*band_ChrI‐8:9 Mb_, where some of the genes were regulated by JU1931, but for many others, a clear pattern was challenging to discern (Figure 3D), indicating that the genetics drivers underlying *trans‐*regulation are complex.

**FIGURE 3:**
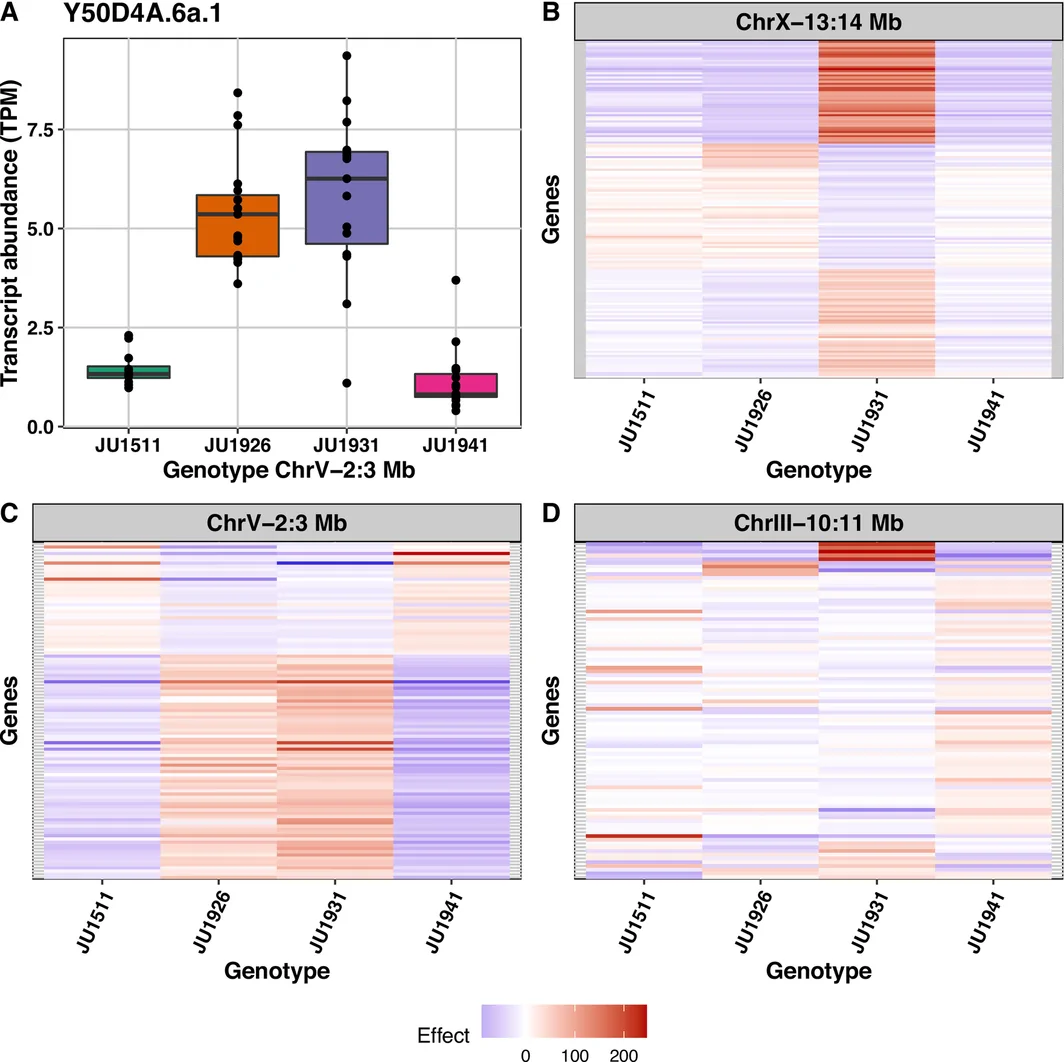
Different trans‐band gene expression dynamics per trans‐band. (A) The expression levels of gene Y50D4A.6a.1 that has an eQTL mapped to ChrV‐2:3 Mb. We observed that gene expression is higher when strains have a JU1926 or JU1931 genotype at location ChrV‐2:3 Mb and a lower gene expression when strains have a JU1511 or a JU1941 genotype at this location. (B–D) Every horizontal line represents the gene expression of an eQTL that mapped to that location. We clustered the effects of the eQTL, according to their underlying genotype at the physical location of the trans‐band, using k‐means clustering. For example: a red color indicates relatively high expression and a blue color indicates a relatively low expression of the eQTL.

### 3.3 *Trans‐*Bands in Response to EtOH Are Associated With Genes Known to Affect EtOH Response Behaviors in Worms

To investigate the biological processes governed by eQTL within the *trans‐*bands, we conducted a GO‐term enrichment analysis on genes featuring eQTL mapped to each *trans‐*band individually. In addition, we sought to assess the relevance of eQTL in the behavioral responses to EtOH by assembling a list of genes that have been experimentally verified to affect EtOH response behaviors in worms and genes that are strongly associated with EtOH use in humans (Piñero et al. 2020; Sterken et al. 2021). We performed a GO‐term enrichment analysis on this dataset (Table S10a). Subsequently, we utilized Cytoscape to analyze GO terms per *trans‐*band, examine the overlap of biological processes across *trans‐*bands, and investigate whether biological processes involved in EtOH response behaviors coincided with GO terms in our *trans‐*bands (Figure 4; Table S10b) (Merico et al. 2010; Shannon et al. 2003).

**FIGURE 4:**
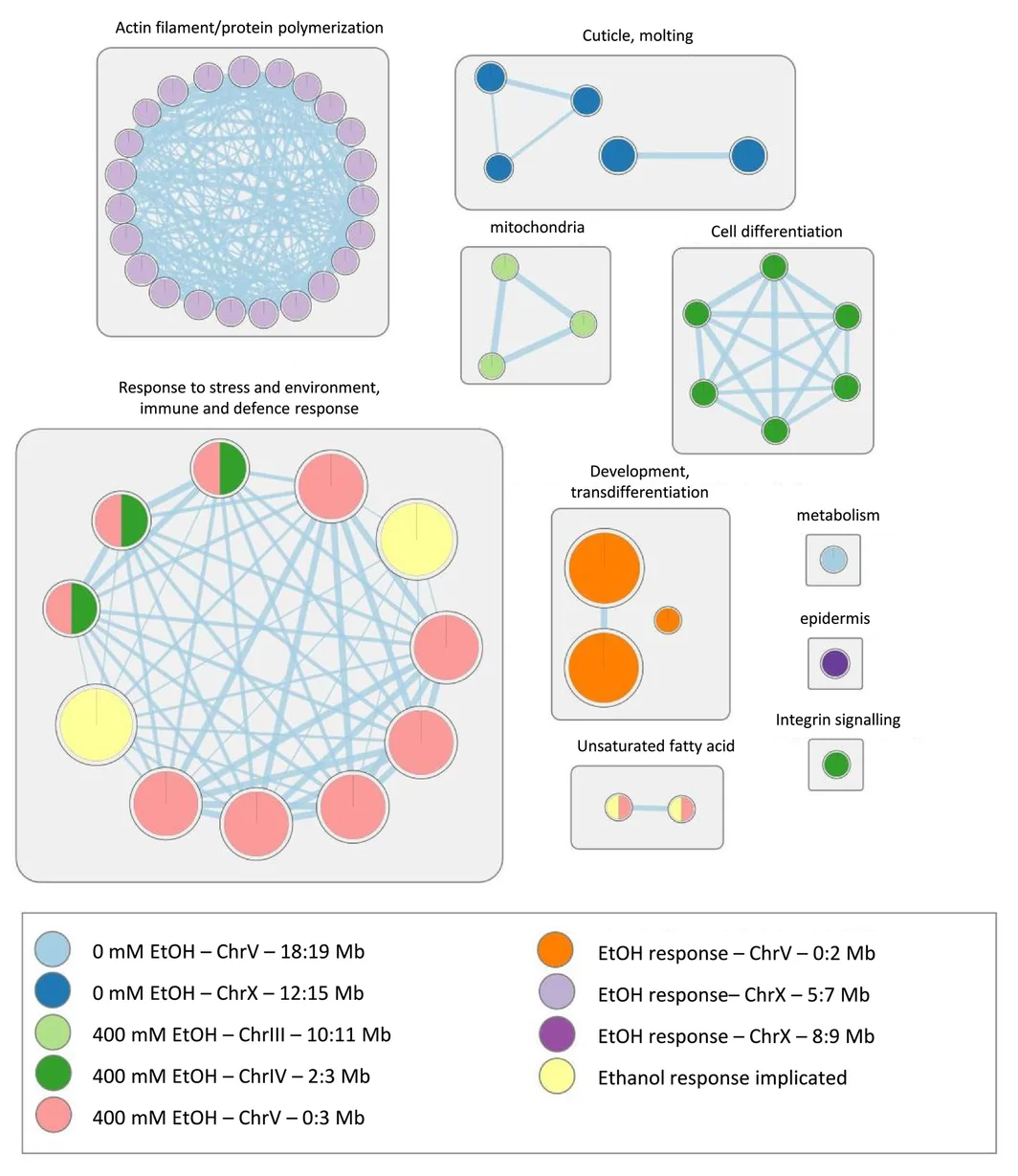
GO‐term enrichment network of genes with an eQTL in trans‐bands. Node size represents the number of genes that fall into a specific enrichment term. Edges between the nodes represent the number of genes that overlap between nodes. The colors of the nodes represent the dataset that was used for the enrichment. Only biological processes were used.

Not every *trans‐*band yielded GO‐term enrichment results. Among the six *trans‐*bands in the 0 mM EtOH condition, only two displayed enrichment for specific biological processes: the cuticle and molting (Chromosome V‐0:2 Mb) and metabolism (Chromosome X‐12:15 Mb) (Figure 4). Biological processes in the EtOH‐responsive gene set included development and transdifferentiation (Chromosome V‐0:2 Mb), epidermis (Chromosome X‐8:9 Mb), and actin filament and protein polymerization (Chromosome X‐5:7 Mb). *Trans‐*bands in the 400 mM EtOH condition exhibited enriched biological processes linked to mitochondria (Chromosome III‐10:11 Mb), cell differentiation and integrin signaling (Chromosome IV‐2:3 Mb). Furthermore, enriched GO terms for stress, environmental, immune, and defense responses, as well as unsaturated fatty acids, were observed in EtOH *trans‐*bands (Chromosome IV‐2:3 & Chromosome V‐0:3). Genes with eQTL mapped to Chromosome V‐0:3 Mb and Chromosome IV‐2:3 Mb in the EtOH condition were associated with biological processes enriched in terms that include known AUD‐implicated genes. This suggests that eQTL within these specific enrichment terms could influence EtOH responses in *C. elegans* .

### 3.4 Confirmation of a Trans‐Band on the Left Arm of Chromosome V That Affects EtOH Responsive Gene Expression

The *trans*‐band_ChrV‐0:3_, unique to the EtOH condition, emerged as the most distinctive EtOH‐specific *trans‐*band in this study. To experimentally confirm the statistical association between the genomic loci on chromosome V‐0:3 Mb and the mapped eQTL, we generated an introgression line (WN1200) using a JU1941 genetic background with a JU1931 introgression locus on chromosome V‐0:3 Mb. In the eQTL analysis, genes mapping to this region exhibited the highest expression when the genomic loci had a JU1931 genetic background and the lowest expression when a JU1941 genetic background was present at those loci (Figures 3B and S4). By selecting the two genotypes with the most extreme phenotypes, we aimed to maximize the impact of genotype on gene expression. WN1200, JU1931, and JU1941 were exposed to 0 mM and 400 mM EtOH, and the introgression on chromosome V was confirmed using the SNP data (Figure S6). However, a genomic trace of WN1158, the starting strain for introgression line generation, was observed at the end of chromosome X (Figure S6).

Subsequently, we correlated the eQTL effect sizes with the gene expression effect sizes in JU1931, JU1941, and the introgression line WN1200. We anticipated positive correlations between JU1931 eQTL effects and parental JU1931 gene expression effects; between JU1941 eQTL effects and parental JU1941 gene expression effects; between JU1931 eQTL effects and WN1200 gene expression effects. We anticipated a negative correlation between JU1941 eQTL effects and WN1200 gene expression effects. In all comparisons, there was a robust correlation of the *cis‐*eQTL in the expected direction (Pearson correlation > 0.8, *p* < 1 × 10^−12^), indicating replicability of these eQTL effects in the single genetic background strains and in the introgression line WN1200 (Figure 5A–D). Although the correlation between JU1931 eQTL effects and parental JU1931 gene expression effects was not as expected (Figure 5A), we successfully validated the *trans‐*eQTL of the *trans‐*band_ChrV‐0:3 Mb_ using other strain comparisons (Pearson correlation > 0.55, *p* < 1 × 10^−15^) (Figure 5B–D). The statistical significance and high correlation between the eQTL and the single backgrounds, as well as the introgression line WN1200 gene expression, strongly support the validity of the mapped *trans‐*band_ChrV‐0:3 Mb_, suggesting the presence of at least one modifier gene in that region regulating the gene expression of associated genes.

**FIGURE 5:**
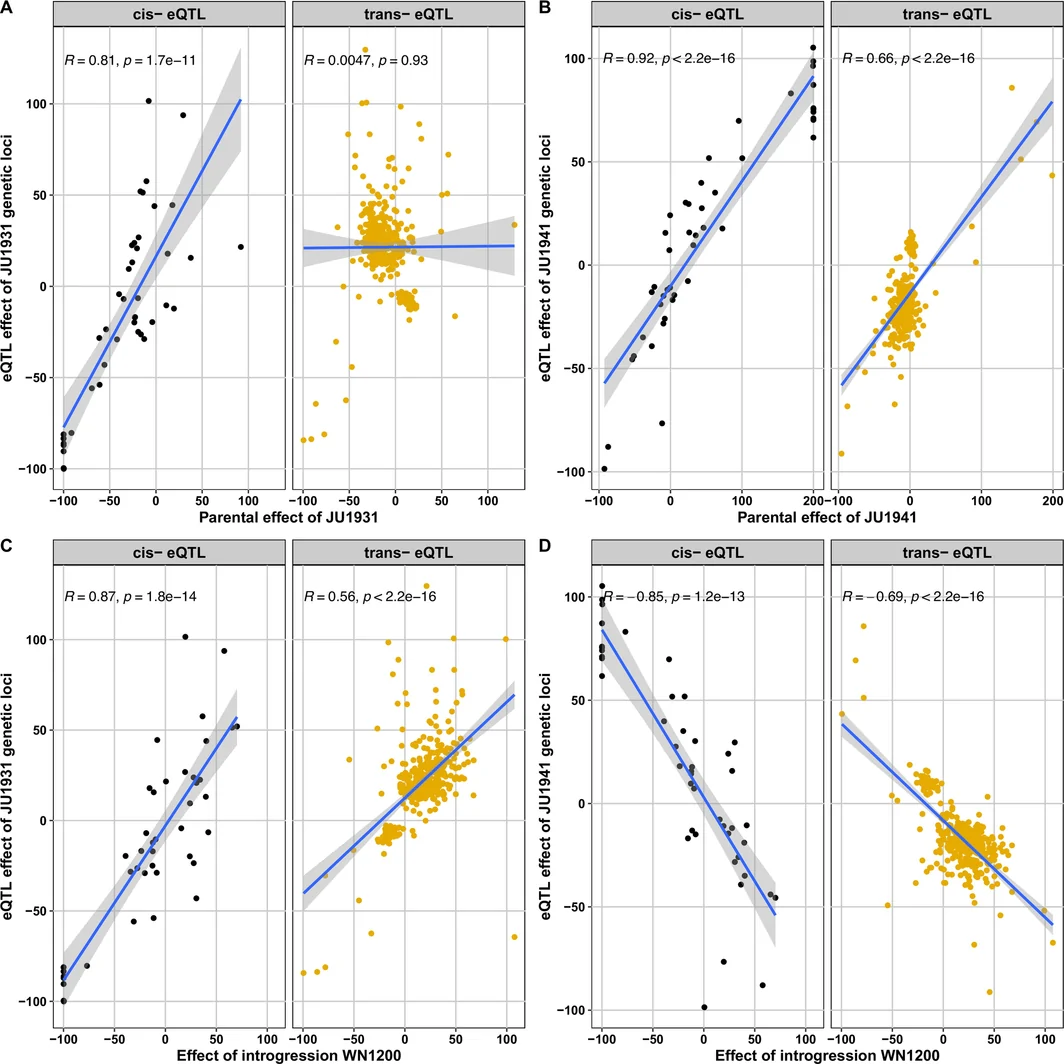
Correlation analysis between the eQTL effects as measured in the mpRILs and the gene expression effects of the introgression validation experiment. Only the eQTL that were mapped in trans‐band_ChrV‐0:3 Mb_ were used in this analysis. WN1200 is an IL with a JU1941 genetic background with a JU1931 introgression at chromosome V‐0:3 Mb. (A) Comparison of eQTL effects of mpRILs that have a JU1931 genotype at trans‐band_ChrV‐0:3 Mb_ and gene expression effects of JU1931 as measured in the IL validation experiment. Each dot represents a gene with an eQTL. (B) Comparison of eQTL effects of mpRILs that have a JU1941 genotype at trans‐band_ChrV‐0:3 Mb_ and gene expression effects of JU1941 as measured in the IL validation experiment. (C) Comparison of eQTL effects of mpRILs that have a JU1931 genotype at trans‐band_ChrV‐0:3 Mb_ and gene expression effects of WN1200 as measured in the IL validation experiment. WN1200 is an IL with a JU1941 genetic background with a JU1931 introgression at chromosome V‐0:3 Mb. (D) Comparison of eQTL effects of mpRILs that have a JU1941 genotype at trans‐band_ChrV‐0:3 Mb_ and gene expression effects of WN1200 as measured in the IL validation experiment.

### 3.5 The Genes *nhr‐131* and *dod‐19* Are Predicted to Be the Modifier Genes of *Trans‐* Band_ChrV_ _‐0:3 Mb_

To identify potential modifier genes regulating the expression of *trans‐*band_ChrV‐0:3 Mb_, we initiated fine mapping by dividing this 3 Mb region into 0.1 Mb bins. We then conducted a correlation analysis on each bin individually, anticipating weaker correlation effects in the first Mb of chromosome V compared to further bins due to less pronounced eQTL effects in the early *trans‐*band segment (Figure S7). As expected, we found stronger and more significant correlations between eQTL and WN1200 gene expression in the region chromosome V‐1:3 Mb compared to the region chromosome V‐0:1 Mb (Figure S7). Consequently, we focused our investigations on the genomic interval chromosome V‐1:3 Mb and the associated eQTL for modifier gene exploration.

We used mediation analysis, the tools Wormnet V3 and Wormexp (Table S12), and comparison between previous eQTL studies to predict modifier genes for *trans‐*band_ChrV‐1:3 Mb_. Utilizing eQTL with the top 20% largest effect sizes in both directions, as measured in WN1200, potential modifier genes were filtered based on their location on chromosome V‐0:3 Mb and the presence of polymorphisms between JU1931 and JU1941. Recognizing biallelic eQTL gene expression dynamics (Figure S4), we prioritized natural variation that was similar in JU1511 & JU1941 versus JU1926 & JU1931. From the mediation analysis, four potential modifier genes emerged, representing the top 10% of *cis‐*eQTL significantly mediating at least 33 *trans‐*eQTL used in our selection (Mediation, *p* < 0.05) (Table 2; Figure S8). Leveraging Wormnet V3, a context‐centric prediction tool for identifying key genes modulating physiology within a specific biological context, three potential candidate modifiers were identified (Tables 2 and S11) (Cho et al. 2014). Our analysis of polymorphisms in the parental strains strongly pointed to two top candidates: *nhr‐131* and *dod‐19* (Table 2). Both genes harbor polymorphisms in the same combination as the *trans‐*eQTL effect direction (Table 2; Figure S4), JU1511 & JU1941 versus JU1926 & JU1931. Since both genes are associated with the *trans‐*band_ChrV‐1:3 Mb_, with *nhr‐131* as a *cis‐*eQTL mediating the expression of *trans‐*eQTL in the selection and *dod‐19* predicted as a hub gene by Wormnet V3, both emerge as strong candidate modifier genes.

**TABLE 2:** Predicted modifier genes of *trans*‐band_ChrV‐1:3 Mb_.

Mediation analysis				
Gene	*cis‐*eQTL effect	Polymorphism	Polymorphisms	Gene function
*nhr‐131*	JU1511 & JU1941 versus JU1926 & JU1931	JU1511 & JU1941 versus JU1926 & JU1931	SNP in 5′ UTR	DNA‐binding transcription factor activity
*srp‐3*	JU1511 & JU1931 versus JU1926 & JU1941	JU1511 & JU1931 versus JU1926 & JU1941	SNP in 5′ UTR, 42V > 42A,118L > 118M, 240N > 240D, SNP in 3′ UTR	Predicted to be involved in proteolysis
*nhr‐110*	JU1511 & JU1931 versus JU1926 & JU1941	JU1511 & JU1931 versus JU1926 & JU1941	SNP in 5′ UTR, 52I > 52F, 87T > 87A, 185T > 185A	DNA‐binding transcription factor activity
*T10B5.7*	JU1511 & JU1941 versus JU1926 & JU1931	JU1511 & JU1931 versus JU1926 & JU1941	24D > 24N	Predicted to be involved in lipid metabolic process

Wormnet V3			
Gene	Polymorphism	Polymorphisms	Gene function
*folt‐2*	JU1931 versus JU1511 & JU1926 & JU1941	227M > 227I, 305A > 305V, 423T	Predicted to be involved in transmembrane transport
*dhs‐14*	JU1931 versus JU1511 & JU1926 & JU1941	43L > 43I, SNP in 3′ UTR	Predicted to enable oxidoreductase activity.
*dod‐19*	JU1511 & JU1941 versus JU1926 & JU1931	316W > 316C, 163W > 163C, 275P > 275S, 122P > 122S, 188L > 188P	Membrane bound, found in lipid raft. Implicated in the innate immune response

Wormexp			
Gene	Polymorphism	Polymorphisms	Gene function
*nuo‐5*	JU1931 versus JU1511 & JU1926 & JU1941	151G, 216F, 379G > 379S, 674P > 674T, snp in 3′ UTR	Predicted to contribute to NADH dehydrogenase activity.

*Note:* Top candidates are highlighted in green.

### 3.6 *dod‐19* Modulates the Development of Acute Functional Tolerance to EtOH

We reasoned that important regulators of gene expression in response to EtOH are likely to influence the acute physiological response to EtOH, and that strong changes in their function may yield EtOH response phenotypes. To test this hypothesis, we obtained predicted null mutants for both *nhr‐131* and *dod‐19* and tested their acute EtOH response behaviors (Figure 6). These mutations reside in the standard laboratory wild‐type strain Bristol N2, so we examined the effects of the mutations relative to the wild‐type N2 background. We found that the acute EtOH responses of *nhr‐131(tm1376)* mutant animals were not different from N2. In contrast, we found that *dod‐19(ok2679)* mutant animals had significantly more development of AFT than N2, indicating that the function of *dod‐19* is important for limiting the development of AFT to EtOH.

**FIGURE 6:**
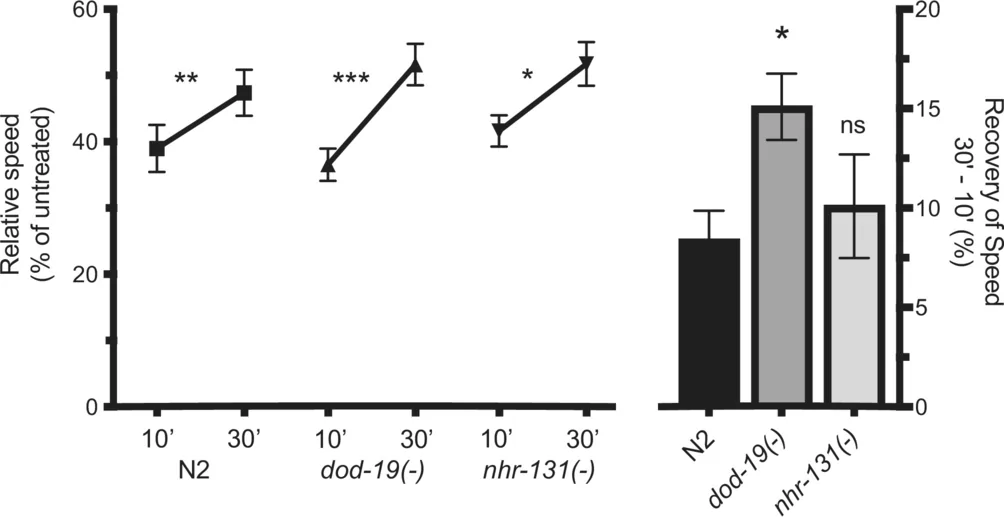
The lipid raft‐resident membrane protein DOD‐19 modulates the rate of the development of AFT. Animals were tested on 0 mM and 400 mM exogenous EtOH. Relative speeds were calculated as treated over untreated speeds at 10 and 30 min of exposure to EtOH (left graph). AFT is the recovery of speed between 10 and 30 min of continuous exposure to EtOH (right graph). N2, *dod‐19(ok2679)* and *nhr‐131(tm1376)* were all tested simultaneously on the same plates. Wild‐type N2 and *nhr‐131* mutant animals develop equivalent AFT (N2 vs. *nhr‐131*, *t*_10_ = 0.57, *p* = 0.58 with an effect size (Cohen's *d*) of 0.33); *dod‐19* mutant animals develop significantly more AFT than N2 with the same exposure to EtOH (N2 vs. *dod‐19*, t_10_ = 3.015, *p* = 0.013 with an effect size (Cohen's *d*) of 1.74). Error bars indicate S.E.M. Paired two‐tailed Student's *t*‐tests were used for statistical comparisons of initial sensitivity and the development of AFT (left graph), unpaired two‐tailed *t*‐tests were used for statistical comparisons of degrees of AFT (right graph). For indicated comparisons: ns, not significant; *p ≤ 0.05; **p ≤ 0.001; ***p ≤ 0.0001.

## 4 Discussion

### 4.1 The Regulation of Gene Expression Shifts in Response to EtOH

We examined the effect of naturally occurring genetic variation on gene expression regulation in response to EtOH in *C. elegans* . We subjected 70 mpRILs and their wild‐type parents (JU1511, JU1926, JU1931, and JU1941) to 0 mM and 400 mM EtOH, followed by whole‐animal transcriptome RNA‐sequencing and eQTL mapping. This allowed us to statistically associate polymorphic loci with individual variations in gene expression. Gene expression can be regulated locally (*cis‐*eQTL), such as by a polymorphism in the promoter sequence of the gene, or distantly (*trans‐*eQTL), for instance, by a transcription factor. Previous eQTL investigations across diverse organisms and experimental conditions consistently underscore the prominence of *trans‐*eQTL in responding to environmental changes. In *C. elegans* , exposure to distinct temperatures primarily induces differences in *trans‐*regulation (Snoek et al. 2017). Furthermore, when *Drosophila melanogaster* was exposed to 10% (v/v) EtOH, the majority of EtOH‐specific eQTL were found in *trans* (Morozova et al. 2022). Recently *trans*‐eQTL in yeast were scrutinized for their role in regulating complex traits in relation to the environment. It was suggested that trans‐eQTL hotspots emanate from genetic variants that affect critical cell states where they functionally converge throughout the genome. Environmentally challenged cells, for example, by abiotic or chemical stress, may provide a genetic state that facilitates resilience to stress regulated by trans‐eQTL (Renganaath and Albert 2025).

Consistent with these observations, our study revealed a substantial shift in *trans‐*eQTL between the control and EtOH‐treated conditions. The overlap of *trans‐*eQTL was considerably lower compared to *cis‐*eQTL overlap (8.1% overlapping *trans‐*eQTL versus 45.1% overlapping *cis‐*eQTL) between the control and EtOH‐treated conditions. A larger overlap of *cis‐*eQTL as opposed to *trans‐*eQTL between different perturbations has been observed in *C. elegans* before; when animals were exposed to three distinct temperature regimes, an overlap of 61% was observed among genes exhibiting a *cis‐*eQTL between two or all three treatments, contrasting sharply with 14% overlap observed among genes with a *trans‐*eQTL under the same condition (Snoek et al. 2017). Notably, we detected more *trans‐*eQTL in the EtOH‐treated condition (2679 *trans‐*eQTL) compared to the control condition (1558 *trans‐*eQTL), which cannot be attributed to differences in statistical power, given that these are the same populations of animals tested on 0 mM and 400 mM EtOH in parallel. This trend of increased *trans‐*eQTL prevalence in perturbed environments aligns with prior observations, such as in the context of genetic perturbation versus control conditions and control versus heat stress (Snoek et al. 2017; van Wijk et al. 2023).

### 4.2 Prominent Role of the First Three Mb of Chromosome V in the Regulation of EtOH Response Genes

When an excess of *trans‐*eQTL converges on the same genomic location, surpassing the occurrence we expect by chance, we categorize these regions as hot spot regions, also recognized as *trans‐*bands. The genomic domains underlying these *trans‐*bands are likely to harbor polymorphic modifier genes that have regulatory control over the expression of numerous genes. Notably, we identified a distinctive EtOH‐specific *trans‐*band on the left arm of chromosome V, that appears to regulate 354 *trans‐*eQTL. Genes associated with eQTL in this *trans‐*band exhibited enrichment in immune and defense responses, responses to stress and environment, and unsaturated fatty acids. Our previous work has demonstrated the significance of lipid composition for EtOH responses in *C. elegans*, mice, and humans (Aliev et al. 2022; Bettinger et al. 2012; Edwards et al. 2019; Raabe et al. 2014; Wolstenholme et al. 2018). Specifically, we have found that genetic mutations that prevent the formation of the long‐chain omega‐3 polyunsaturated fatty acid eicosapentaenoic acid (EPA) result in animals being unable to develop acute functional tolerance to EtOH (Raabe et al. 2014). Interestingly, we identified eQTL in the EtOH condition that mapped to the *trans‐*band_Chr V‐0:3 Mb_ for the *fat‐1*, *fat‐3*, and *fat‐4* genes encoding enzymes in the biosynthetic pathway for long‐chain omega‐3 fatty acids. Natural variation in fatty acid metabolites has also been observed within a RIL panel derived from two other genetically divergent wild strains, N2 and CB4856, supporting the possibility that there may be natural genetic variation in fatty acid composition between the mpRILs, potentially leading to differential EtOH response behaviors (Gao et al. 2018).

To confirm the significance of this prominent ethanol *trans‐*band on chromosome V, we engineered an introgression line (IL) in which the left arm of chromosome V contains the JU1931 genotype, whereas the remainder of the genome has the JU1941 genetic background. We selected these two genotypes based on the robust regulation of gene expression in the opposite direction observed in animals with either JU1931 or JU1941‐like genomic loci at this *trans‐*band mapping location. Correlation analysis of the eQTL effect and gene expression effect in WN1200 validated the existence of this *trans‐*band. Thus, we infer that the first 3 Mb of chromosome V regulates the gene expression of numerous genes involved in EtOH‐related processes. The use of ILs for *trans‐*band validation in *C. elegans* has been explored previously, and very similar Pearson correlations have been detected; where Sterken et al. (2020) noted an average Pearson correlation of 0.66, we observed an average Pearson correlation of 0.67.

The intricate nature of *trans‐*eQTL becomes evident in studies on polymorphic loci regulation, as exemplified in the yeast *Saccharomyces cerevisiae* (Lutz et al. 2022). Here, the regulation of *trans‐*eQTL can be traced to specific (interacting) nucleotides within a gene, showcasing the additive and epistatic effects on gene expression. It is noteworthy that ILs may not consistently replicate results obtained from recombinant inbred lines or chromosome substitution strain experiments. We have successfully mapped two additive behavioral QTL in an mpRIL panel examining locomotion behavior in response to low EtOH concentrations, yet these findings failed replication in ILs. Similar challenges were observed in lifespan assays between N2 and CB4856 genotypes (Doroszuk et al. 2009; Glauser et al. 2011). Although a genome‐wide IL panel identified QTL affecting lifespan on chromosomes I, II, III, and IV (Doroszuk et al. 2009), chromosome substitution strains only revealed a pronounced effect on lifespan on chromosome X (Glauser et al. 2011). These examples underscore the intricate nature of quantitative traits, emphasizing that the absence of replication does not necessarily negate the existence of QTL but can also suggest more complex underlying mechanisms.

### 4.3 *dod‐19* Negatively Regulates the Development of AFT to EtOH

We narrowed down potential modifier genes underlying *trans*‐band_ChrV‐0:3_ by employing mediation analysis, Wormnet V3, Wormexp, and comparison with other eQTL studies. We identified *nhr‐131* and *dod‐19* as strong candidate genes for regulating the gene expression response to EtOH. *nhr‐131* encodes a nuclear hormone receptor with homology to the nuclear hormone receptor family in mammals, whereas *dod‐19* encodes a membrane glycoprotein without an obvious mammalian ortholog. We predicted that these genes may therefore be important for controlling the normal physiological response to EtOH. To test this hypothesis, we examined strong loss‐of‐function mutations in these genes for acute EtOH response behaviors. Although inactivation of *nhr‐131* did not yield a detectable change in EtOH response phenotypes, animals carrying a null mutation in *dod‐19* demonstrated a strong enhancement of AFT. This supports a role of *dod‐19* in influencing gene expression that is required for limiting the development of AFT. *dod‐19* is predicted to encode a membrane‐bound protein that has been found in lipid raft microdomains (Rao et al. 2011). Intriguingly, we previously found that loss of the lipid‐regulating triacylglyceride lipase, LIPS‐7, also enhances AFT, and that this alters the function of the EtOH‐responsive membrane channel SLO‐1 (Bettinger et al. 2012), which is known to be found in lipid rafts (Lee et al. 2021; Weaver et al. 2007). Together, these results suggest a model in which AFT requires *dod‐19* and *lips‐7* regulation of the lipid microarchitecture of the plasma membrane.

## Author Contributions

**Marijke H. van Wijk:** experimental design, experimental work, data analysis, manuscript preparation, key discussions. **Mark G. Sterken:** experimental design, data analysis, key discussions, manuscript preparation. **Elizabeth C. Quamme:** *C. elegans* exposure to ethanol. **Joost A. G. Riksen:** experimental work. **Basten L. Snoek:** key discussions. **Stefan J. S. van de Ruitenbeek:** RNA alignment to reference genome and SNP calling. **Laura D. Mathies:** *C. elegans* behavioral studies. **Andrew G. Davies:** experimental design, key discussions. **Jill C. Bettinger:** experimental design, manuscript preparation, key discussions. **Jan E. Kammenga:** experimental design, key discussions, manuscript preparation. All authors read and approved the final manuscript.

## Funding

This work was supported by the National Institute on Alcohol Abuse and Alcoholism (R01 AA026658) and Nederlandse Organisatie voor Wetenschappelijk Onderzoek (Grant 17282).

## Conflicts of Interest

The authors declare no conflicts of interest.

## Supporting information


**Figure S1:** Selection of the core mpRIL population for expression analysis. For each subset size 1000,000 random populations were created and genetic correlations were calculated based on pairwise SNPs correlation. The median correlation of a population was classified as the population correlation and was used for further analysis. (A) Genetic correlation of subset size and type. As expected, the differences in genetic correlations between the selected populations became smaller as the subset size grew bigger. (B) The allele frequency between the selected subsets that were used in the power simulations were stable, with variability only in subsets that were smaller than 50 strains. (C) Single marker power simulations show increasing true QTL detection with increasing subset size. Furthermore, subsets with a low population correlation per subset size outperformed subsets with a high population correlation. (D) Genetic map of the lowest correlating population consisting of the 70 strains that we chose for eQTL mapping. This population has the power to detect 82% of eQTL that explain at least 35% of the variance based on a single marker SNP mapping. (E) Allele frequencies of the mapping population show a consistent distribution of genotypes.

**Figure S2:** Distinct biological processes are regulated upon EtOH exposure. Node size represents the number of genes that fall into a specific enrichment term. Edges between the nodes represent the number of genes that overlap between nodes. The colors of the nodes represent the dataset that was used for the enrichment: upregulated genes in response to ethanol (red) and downregulated genes in response to ethanol (blue). Only GO terms associated with biological processes were used.

**Figure S3:** Different trans‐band gene expression dynamics per trans‐band in the 0 mM EtOH condition. Every horizontal line represents the gene expression of an eQTL that mapped to that location. The effects of the eQTL according to their underlying genotype on the physical location of the trans‐band were clustered by k‐means clustering.

**Figure S4:** Different trans‐band gene expression dynamics per trans‐band in the 400 mM EtOH condition. Every horizontal line represents the gene expression of an eQTL that mapped to that location. The effects of the eQTL according to their underlying genotype on the physical location of the trans‐band were clustered by k‐means clustering.

**Figure S5:** Different trans‐band gene expression dynamics per trans‐band in the EtOH response condition. Every horizontal line represents the gene expression of an eQTL that mapped to that location. The effects of the eQTL according to their underlying genotype on the physical location of the trans‐band were clustered by k‐means clustering.

**Figure S6:** Genetic map of the introgression line (IL) WN1200. The genetic background of WN1200 is JU1941‐like and has the desired JU1931 introgression on chromosome V‐0:3 Mb. Note that at the end of chromosome X there is a relic from the starting strain WN1158.

**Figure S7:** Fine mapping of *trans‐*band_ChrV‐0:3 Mb_. The three Mb spanning the locus was divided into bins of 0.1 Mb and the correlations between the eQTL effects as measured in the mpRILs and the gene expression effects of the introgression validation experiment were calculated. The color represents the Pearson correlation and the numbers in black are the *p* values.

**Figure S8:** The results of the mediation analysis. We tested if *cis‐*eQTL mapping to *trans‐*band_ChrV‐0:3 Mb_ were mediating between the genotype on chromosome V‐0:3 Mb and *trans‐*eQTL in *trans‐*band_ChrV:‐0:3 Mb_.

**Figure S9:** The outcrossing scheme for isolating *nhr‐131(tm1376)* and *dod‐19(ok2679)*. Before behavioral testing, both *dod‐19(ok2679)* and *nhr‐131(tm1376)* were backcrossed to N2 six times. Deletions were detected using PCR with primers flanking the allele. Homozygotes were confirmed by using PCR with a primer that can only amplify from wild‐type DNA.

**Data S1:** Supporting Information.

## Data Availability

Raw data is made publicly available on ArrayExpress under E‐MTAB‐15535. The mapped eQTL can be analyzed on WormQTL2.
