# Supplementary material for: Expression Profiling of a Recombinant Inbred Line Panel Identifies the dod‐19 Gene as a Regulator of Ethanol Response Behaviors in C. elegans
Source: Alcohol Clin Exp Res (Hoboken). 2026 Oct 23;50(10):e70432. doi: 10.1111/acer.70432 (PMC13694738; doi:10.1111/acer.70432)
Supplement: Supplementary file 1 [file ACER-50-0-s002.pdf]

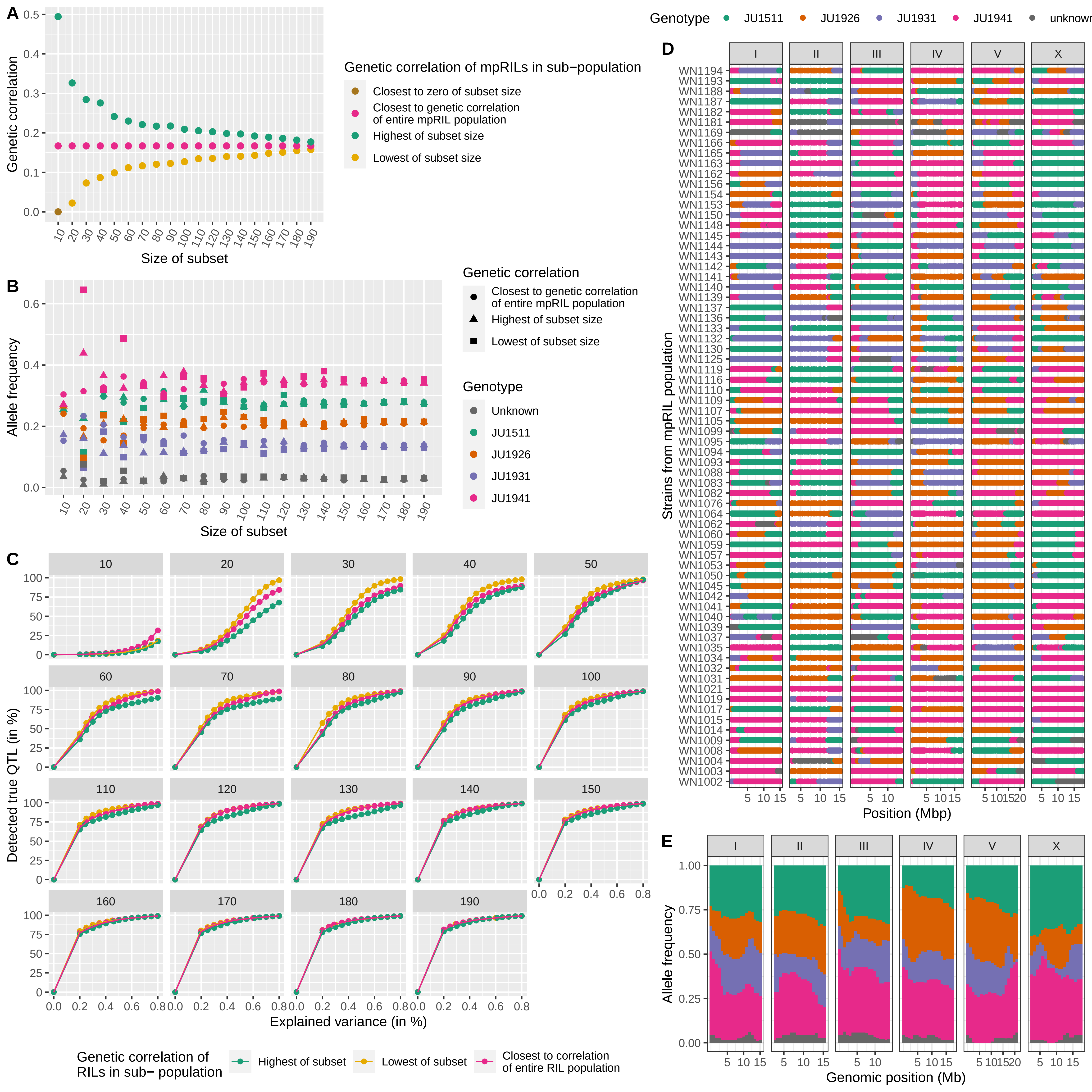

A
Genetic correlation
Size of subset
Genetic correlation of mpRILs in sub−population
Closest to zero of subset size
Closest to genetic correlation of entire mpRIL population
Highest of subset size
Lowest of subset size
B
Allele frequency
Size of subset
Genetic correlation
Closest to genetic correlation of entire mpRIL population
Highest of subset size
Lowest of subset size
Genotype
Unknown
JU1511
JU1926
JU1931
JU1941
C
Detected true QTL (in %)
Explained variance (in %)
Genetic correlation of RILs in sub− population
Highest of subset
Lowest of subset
Closest to correlation of entire RIL population
Genotype
JU1511
JU1926
JU1931
JU1941
unknown
D
I
II
III
IV
V
X
Strains from mpRIL population
WN1194
WN1193
WN1188
WN1187
WN1182
WN1181
WN1169
WN1166
WN1165
WN1163
WN1162
WN1156
WN1154
WN1153
WN1150
WN1148
WN1145
WN1144
WN1143
WN1142
WN1141
WN1140
WN1139
WN1137
WN1136
WN1133
WN1132
WN1130
WN1125
WN1119
WN1116
WN1110
WN1109
WN1107
WN1105
WN1099
WN1095
WN1094
WN1093
WN1088
WN1083
WN1082
WN1076
WN1064
WN1062
WN1060
WN1059
WN1057
WN1053
WN1050
WN1045
WN1042
WN1041
WN1040
WN1039
WN1037
WN1035
WN1034
WN1032
WN1031
WN1021
WN1019
WN1017
WN1015
WN1014
WN1009
WN1008
WN1004
WN1003
WN1002
Position (Mbp)
E
I
II
III
IV
V
X
Allele frequency
Genomic position (Mb)
