# Supplementary material for: Expression Profiling of a Recombinant Inbred Line Panel Identifies the dod‐19 Gene as a Regulator of Ethanol Response Behaviors in C. elegans
Source: Alcohol Clin Exp Res (Hoboken). 2026 Oct 23;50(10):e70432. doi: 10.1111/acer.70432 (PMC13694738; doi:10.1111/acer.70432)
Supplement: Supplementary file 2 [file ACER-50-0-s003.pdf]

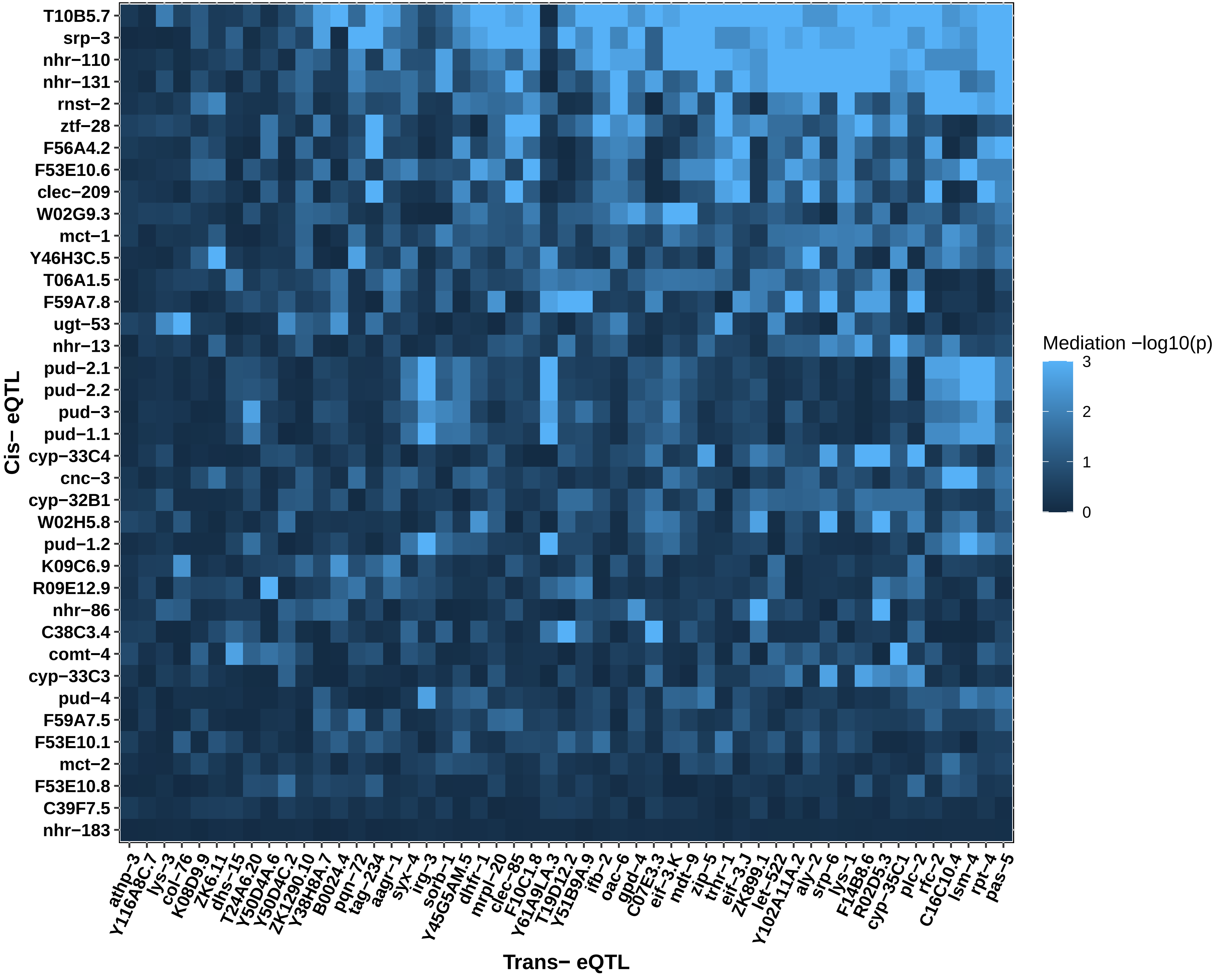

T10B5.7
srp−3
nhr−110
nhr−131
rnst−2
ztf−28
F56A4.2
F53E10.6
clec−209
W02G9.3
mct−1
Y46H3C.5
T06A1.5
F59A7.8
ugt−53
nhr−13
pud−2.1
pud−2.2
pud−3
pud−1.1
cyp−33C4
cnc−3
cyp−32B1
W02H5.8
pud−1.2
K09C6.9
R09E12.9
nhr−86
C38C3.4
comt−4
cyp−33C3
pud−4
F59A7.5
F53E10.1
mct−2
F53E10.8
C39F7.5
nhr−183
Cis− eQTL
athp−3
Y116A8C.7
lys−3
col−76
K08D9.9
ZK6.11
dhs−15
T24A6.20
Y50D4A.6
Y50D4C.2
ZK1290.10
Y38H8A.7
B0024.4
pqn−72
tag−234
aagr−1
syx−4
irg−3
sorb−1
Y45G5AM.5
dhfr−1
mrpl−20
clec−85
F10C1.8
Y61A9LA.3
T19D12.2
Y51B9A.9
ifb−2
oac−6
gpd−4
C07E3.3
eif−3.K
mdt−9
zip−5
trhr−1
eif−3.J
ZK899.1
let−522
Y102A11A.2
aly−2
srp−6
lys−1
F14B8.6
R02D5.3
cyp−35C1
plc−2
rfc−2
C16C10.4
lsm−4
rpt−4
pas−5
Trans− eQTL
Mediation −log10(p)
3
2
1
0
