# Supplementary material for: Expression Profiling of a Recombinant Inbred Line Panel Identifies the dod‐19 Gene as a Regulator of Ethanol Response Behaviors in C. elegans
Source: Alcohol Clin Exp Res (Hoboken). 2026 Oct 23;50(10):e70432. doi: 10.1111/acer.70432 (PMC13694738; doi:10.1111/acer.70432)
Supplement: Supplementary file 3 [file ACER-50-0-s004.pdf]

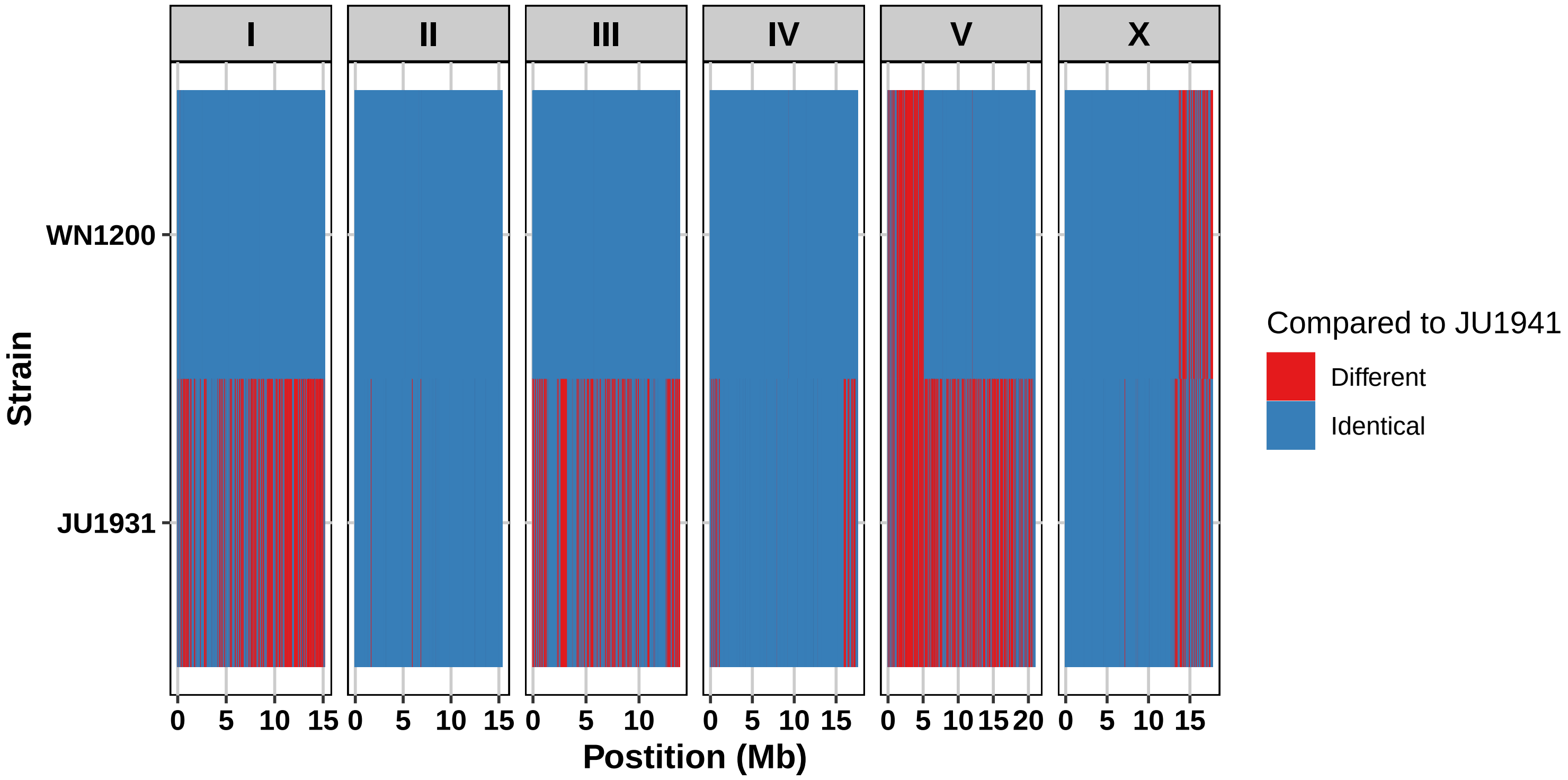

I
II
III
IV
V
X
WN1200
JU1931
Strain
0
5
10
15
20
Postition (Mb)
Compared to JU1941
Different
Identical
