# Supplementary material for: Expression Profiling of a Recombinant Inbred Line Panel Identifies the dod‐19 Gene as a Regulator of Ethanol Response Behaviors in C. elegans
Source: Alcohol Clin Exp Res (Hoboken). 2026 Oct 23;50(10):e70432. doi: 10.1111/acer.70432 (PMC13694738; doi:10.1111/acer.70432)
Supplement: Supplementary file 4 [file ACER-50-0-s005.pdf]

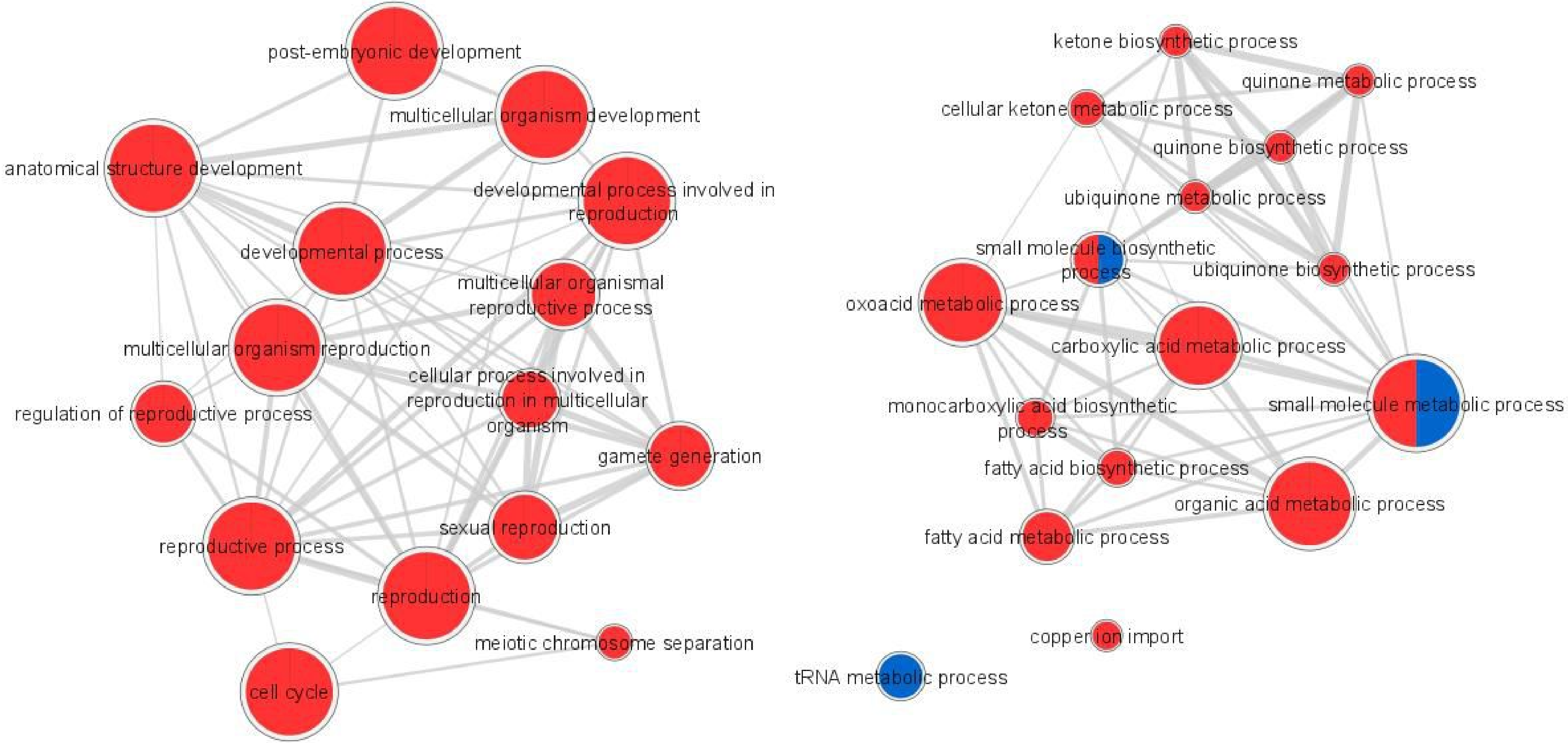
post-embryonic development
multicellular organism development
anatomical structure development
developmental process involved in reproduction
developmental process
multicellular organismal reproductive process
multicellular organism reproduction
cellular process involved in reproduction in multicellular organism
regulation of reproductive process
gamete generation
sexual reproduction
reproductive process
reproduction
meiotic chromosome separation
cell cycle
ketone biosynthetic process
quinone metabolic process
cellular ketone metabolic process
quinone biosynthetic process
ubiquinone metabolic process
small molecule biosynthetic process
ubiquinone biosynthetic process
oxoacid metabolic process
carboxylic acid metabolic process
small molecule metabolic process
monocarboxylic acid biosynthetic process
fatty acid biosynthetic process
organic acid metabolic process
fatty acid metabolic process
copper ion import
tRNA metabolic process
