# Supplementary material for: Expression Profiling of a Recombinant Inbred Line Panel Identifies the dod‐19 Gene as a Regulator of Ethanol Response Behaviors in C. elegans
Source: Alcohol Clin Exp Res (Hoboken). 2026 Oct 23;50(10):e70432. doi: 10.1111/acer.70432 (PMC13694738; doi:10.1111/acer.70432)
Supplement: Supplementary file 5 [file ACER-50-0-s006.pdf]

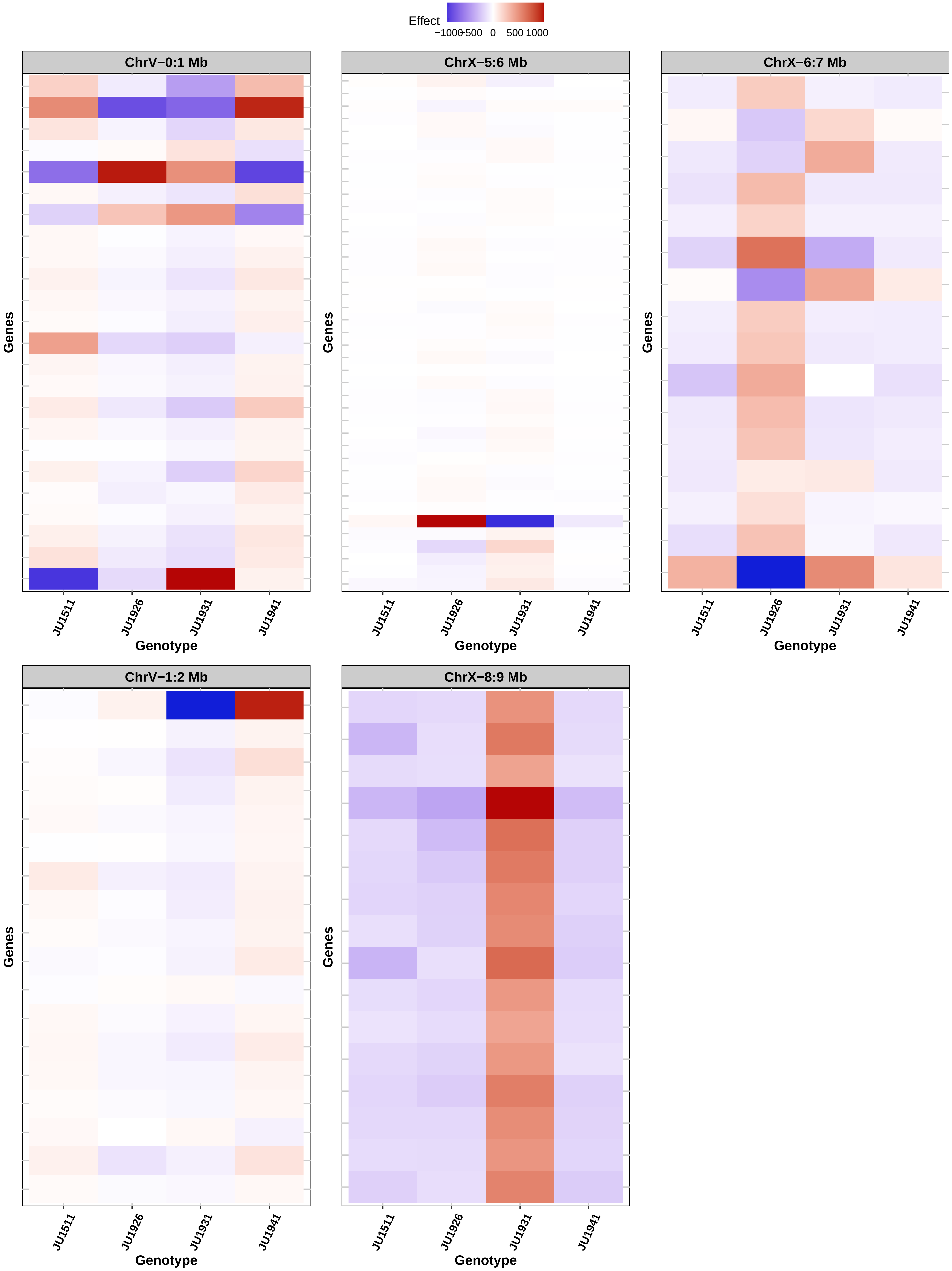

Effect
−1000
−500
0
500
1000
ChrV−0:1 Mb
ChrX−5:6 Mb
ChrX−6:7 Mb
ChrV−1:2 Mb
ChrX−8:9 Mb
Genes
Genotype
JU1511
JU1926
JU1931
JU1941
