# Supplementary material for: Expression Profiling of a Recombinant Inbred Line Panel Identifies the dod‐19 Gene as a Regulator of Ethanol Response Behaviors in C. elegans
Source: Alcohol Clin Exp Res (Hoboken). 2026 Oct 23;50(10):e70432. doi: 10.1111/acer.70432 (PMC13694738; doi:10.1111/acer.70432)
Supplement: Supplementary file 6 [file ACER-50-0-s007.pdf]

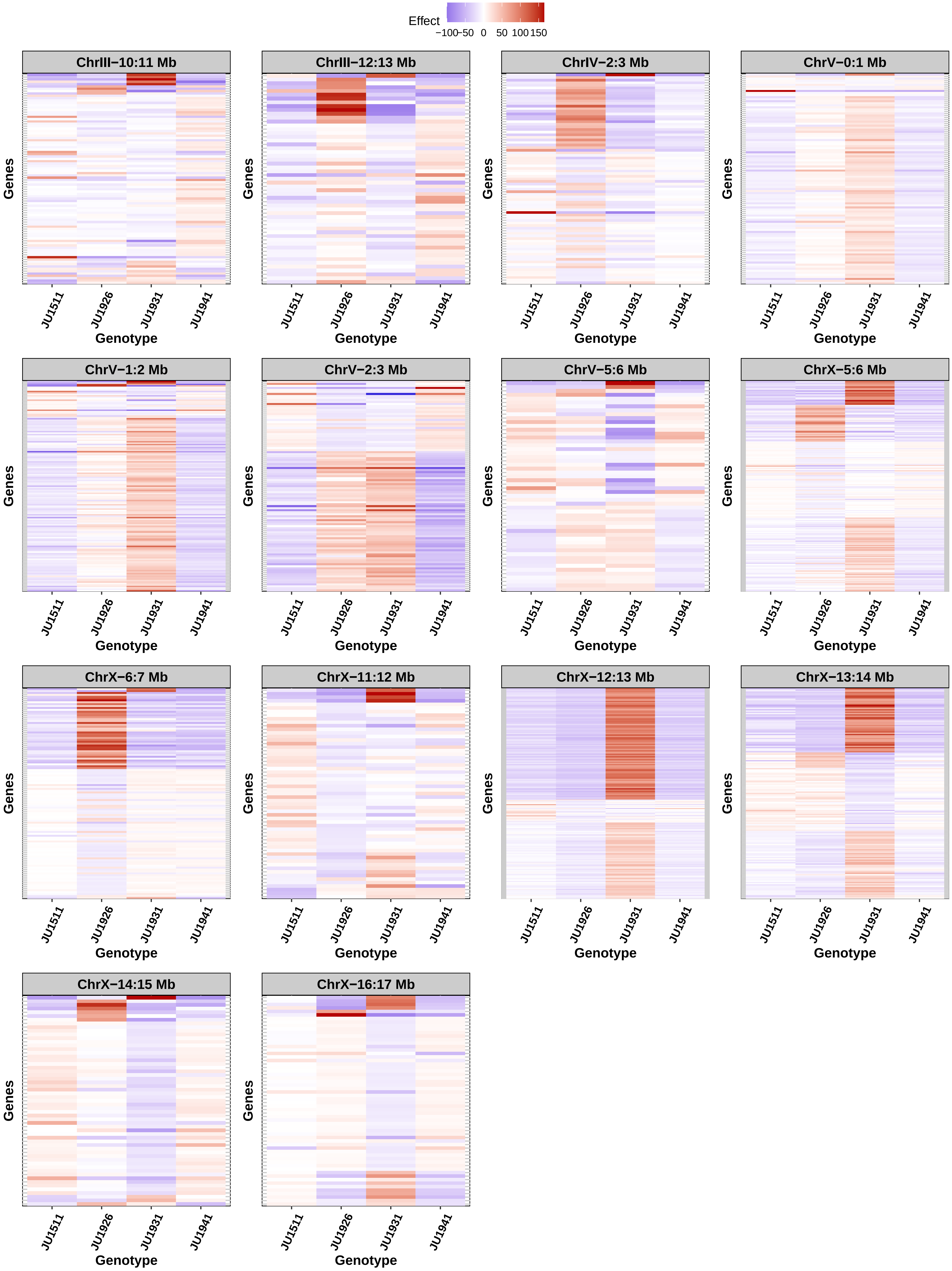
Effect
−100
−50
0
50
100
150
ChrIII−10:11 Mb
ChrIII−12:13 Mb
ChrIV−2:3 Mb
ChrV−0:1 Mb
ChrV−1:2 Mb
ChrV−2:3 Mb
ChrV−5:6 Mb
ChrX−5:6 Mb
ChrX−6:7 Mb
ChrX−11:12 Mb
ChrX−12:13 Mb
ChrX−13:14 Mb
ChrX−14:15 Mb
ChrX−16:17 Mb
Genes
JU1511
JU1926
JU1931
JU1941
Genotype
