# Supplementary material for: Expression Profiling of a Recombinant Inbred Line Panel Identifies the dod‐19 Gene as a Regulator of Ethanol Response Behaviors in C. elegans
Source: Alcohol Clin Exp Res (Hoboken). 2026 Oct 23;50(10):e70432. doi: 10.1111/acer.70432 (PMC13694738; doi:10.1111/acer.70432)
Supplement: Supplementary file 7 [file ACER-50-0-s009.pdf]

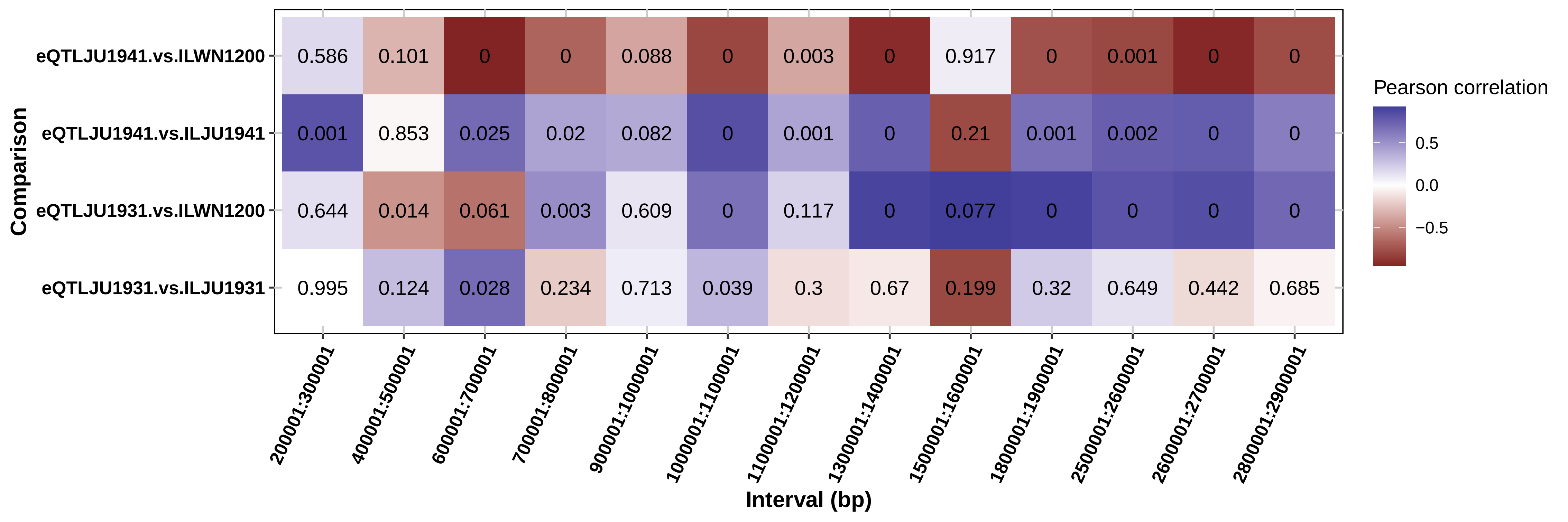

Comparison
eQTLJU1941.vs.ILWN1200
eQTLJU1941.vs.ILJU1941
eQTLJU1931.vs.ILWN1200
eQTLJU1931.vs.ILJU1931
0.586 0.101 0 0 0.088 0 0.003 0 0.917 0 0.001 0 0
0.001 0.853 0.025 0.02 0.082 0 0.001 0 0.21 0.001 0.002 0 0
0.644 0.014 0.061 0.003 0.609 0 0.117 0 0.077 0 0 0 0
0.995 0.124 0.028 0.234 0.713 0.039 0.3 0.67 0.199 0.32 0.649 0.442 0.685
200001:300001
400001:500001
600001:700001
700001:800001
900001:1000001
1000001:1100001
1100001:1200001
1300001:1400001
1500001:1600001
1800001:1900001
2500001:2600001
2600001:2700001
2800001:2900001
Interval (bp)
Pearson correlation
0.5
0.0
−0.5
