# Supplementary material for: Expression Profiling of a Recombinant Inbred Line Panel Identifies the dod‐19 Gene as a Regulator of Ethanol Response Behaviors in C. elegans
Source: Alcohol Clin Exp Res (Hoboken). 2026 Oct 23;50(10):e70432. doi: 10.1111/acer.70432 (PMC13694738; doi:10.1111/acer.70432)
Supplement: Supplementary file 9 [file ACER-50-0-s008.docx]

Before behavioral testing, both *dod-19(ok2679)* and *nhr-131(tm1376)* were backcrossed to N2 six times. Deletions were detected using PCR with primers flanking the allele. Homozygotes were confirmed by using PCR with a primer that can only amplify from wild type DNA.

*nhr-131(tm1376)* has a 325 bp deletion that removes the proximal promoter and first exon of *nhr-131a*, as well as part of exon 2; *nhr-131b* is unaffected. *dod-19(ok2629)* contains a 759 bp deletion that removes sequences from the last three exons of the gene; all isoforms are affected. The *ok2629* deletion is predicted to be a strong loss-of-function for *dod-19.* All primers are listed in Table x.

| **Primer name** | **Primer sequence** | **PCR** | **WT** | **mutant** |
| --- | --- | --- | --- | --- |
| tm1376_F | ATCTGGGTTGCCCAAGAAC | tm1376 F + R | 682 bp | 357 bp |
| tm1376_R | ATCCATCCCAACATCTCGGC | tm1376 F + int | 363 bp | x |
| tm1376_int_R | ACCTGAAAAACGCTGCACAC | ok2629 F + R | 1223 bp | 464 bp |
| ok2629_F | CAAACATGGTCTGTGACGCT | ok2629 F + int | 297 bp | x |
| ok2629_R | CGTCGCCTTCCTGTATGC |  |  |  |
| ok2629_int_del | TCCGCATTTCAGAAGGAGCA |  |  |  |
